# Exploring the molecular mechanisms of increased intensity of pyrethroid resistance in Central African population of a major malaria vector *Anopheles coluzzii*

**DOI:** 10.1111/eva.13641

**Published:** 2024-02-26

**Authors:** Amen N. Fadel, Sulaiman S. Ibrahim, Maurice M. Sandeu, Claudine Grâce Maffo Tatsinkou, Benjamin D. Menze, Helen Irving, Jack Hearn, Sanjay C. Nagi, Gareth D. Weedall, Ebai Terence, Williams Tchapga, Samuel Wanji, Charles S. Wondji

**Affiliations:** ^1^ Center for Research in Infectious Diseases (CRID) Yaoundé Cameroon; ^2^ Department of Microbiology and Parasitology University of Buea Buea Cameroon; ^3^ Department of Biochemistry Bayero University Kano Nigeria; ^4^ Vector Biology Department Liverpool School of Tropical Medicine (LSTM) Liverpool UK; ^5^ Department of Microbiology and Infectious Diseases School of Veterinary Medicine and Sciences University of Ngaoundéré Ngaoundéré Cameroon; ^6^ Centre of Epidemiology and Planetary Health North Faculty Veterinary & Animal Science Scotland's Rural College Inverness UK; ^7^ School of Biological and Environmental Sciences Liverpool John Moores University Liverpool UK

**Keywords:** *Anopheles coluzzii*, Cameroon, insecticide, metabolic, resistance

## Abstract

Molecular mechanisms driving the escalation of pyrethroid resistance in the major malaria mosquitoes of Central Africa remain largely uncharacterized, hindering effective management strategies. Here, resistance intensity and the molecular mechanisms driving it were investigated in a population of *Anopheles coluzzii* from northern Cameroon. High levels of pyrethroid and organochloride resistance were observed in *An. coluzzii* population, with no mortality for 1× permethrin; only 11% and 33% mortalities for 5× and 10× permethrin diagnostic concentrations, and <2% mortalities for deltamethrin and DDT, respectively. Moderate bendiocarb resistance (88% mortality) and full susceptibility to malathion were observed. Synergist bioassays with piperonyl butoxide recovered permethrin susceptibility, with mortalities increasing to 53.39%, and 87.30% for 5× and 10× permethrin, respectively, implicating P450 monooxygenases. Synergist bioassays with diethyl maleate (DEM) recovered permethrin and DDT susceptibilities (mortalities increasing to 34.75% and 14.88%, respectively), implicating glutathione *S*‐transferases. RNA‐seq‐based genome‐wide transcriptional analyses supported by quantitative PCR identified glutathione *S*‐transferase, *GSTe2* (RNA‐seqFC = 2.93 and qRT‐PCRFC = 8.4, *p* < 0.0043) and CYP450, *CYP6Z2* (RNA‐seqFC = 2.39 and qRT‐PCRFC = 11.7, *p* < 0.0177) as the most overexpressed detoxification genes in the pyrethroid‐resistant mosquitoes, compared to mosquitoes of the susceptible Ngousso colony. Other overexpressed genes include P450s, *CYP6M2* (FC = 1.68, *p* < 0.0114), *CYP4G16* (FC = 2.02, *p* < 0.0005), and *CYP4G17* (FC = 1.86, *p* < 0.0276). While high frequency of the 1014F *kdr* mutation (50%) and low frequencies of 1014S (6.61%) and 1575Y (10.29%) were observed, no *ace*‐1 mutation was detected in bendiocarb‐resistant populations, suggesting the preeminent role of metabolic mechanism. Overexpression of metabolic resistance genes (including *GSTe2* and *CYP6Z2* known to confer resistance to multiple insecticides) in *An. coluzzii* from the Sudan Savannah of Cameroon highlights the need for alternative management strategies to reduce malaria burden in northern Cameroon.

## 1 INTRODUCTION

Malaria killed 534,000 individuals worldwide in 2019, 95% of which occurred in Africa (WHO, 2021). Insecticide‐based vector control, through sustained distribution of the long‐lasting insecticidal nets (LLINs) and indoor residual spraying (IRS), is the mainstay of malaria control (WHO, 2019) and is credited with reducing malaria morbidity in Africa by an estimated 68% for LLINs and 13% for IRS between 2000 and 2015 (Bhatt et al., 2015; WHO, 2018). However, widespread insecticide resistance in the main malaria vectors is threatening the effectiveness of these control measures (Hemingway et al., 2016; Ranson & Lissenden, 2016). There is concern that escalation in pyrethroid resistance in the major malaria vectors (*An. gambiae* sensu lato and *Anopheles funestus* sensu stricto) could endanger malaria control efforts in Africa (Ibrahim, Fadel, Tchouakui, et al., 2019; Menze et al., 2018; Riveron et al., 2019; Riveron, Tchouakui, et al., 2018). To achieve malaria elimination from endemic regions, data‐informed policymaking by malaria control programs requires temporal and spatial surveillance of insecticide resistance (Churcher et al., 2017).

In Gounougou (Guinea Savannah of northern Cameroon), pyrethroid and DDT resistance (and carbamate and organophosphate susceptibility) were identified in *An*. *coluzzii* populations collected in 2017 (Fadel et al., 2019). However, resistance intensity was not characterized in this study. Measuring resistance intensity, as recommended by the WHO, using 5× and 10× the diagnostic concentrations of pyrethroids (WHO, 2016), allows assessing the extent of resistance in a population and its likely impact on the effectiveness of control tools. Assessing the intensity of resistance is current WHO‐recommended practice (WHO, 2016) because resistance phenotypes detected using the conventional discriminating concentrations do not necessarily provide information relevant to the efficacy failure of that insecticide in the field (Bagi et al., 2015). Measurement of resistance intensity is increasingly being used to quantify the strength of resistance (Awolola et al., 2018; Ibrahim, Fadel, Tchouakui, et al., 2019; Silva et al., 2020). Considering the variation of resistance profiles in *An*. *gambiae* s.l. populations across Cameroon (Antonio‐Nkondjio et al., 2017, 2019), routine monitoring of insecticide resistance in the northern part of the country is necessary to capture the dynamics and evolution of resistance for effective management. Our previous study using synergist bioassays with piperonyl butoxide (PBO) has implicated cytochrome P450 monooxygenases in pyrethroid resistance (Fadel et al., 2019), but the specific genes driving the resistance were not identified.

To address this knowledge gap, we assessed the extent and molecular drivers of pyrethroid resistance escalation in *An*. *coluzzii* populations from Gounougou, northern Cameroon, using 5×and 10× discriminating doses of permethrin, followed by transcriptomic analyses. Synergist bioassays with PBO and diethyl maleate (DEM) were performed to assess the contribution of cytochrome P450 monooxygenases (CYP) and glutathione *S*‐transferases (GST), respectively, to the resistance. Genome‐wide transcriptomic and population genetic analyses using RNA‐seq, supported by qRT‐PCR, identified potential genes contributing to metabolic resistance, including from the CYP and GST gene families. Specifically, *CYP6Z2* and *GSTe2* recently described as two key *An. coluzzii* genes driving resistance in Sahelo‐Sudanian regions of several countries (Ibrahim et al., 2023) were shown to be the most overexpressed in the Gounougou population. In addition, sensory appendage protein (SAP) genes, shown to mediate resistance by reduced uptake of insecticides from *Anopheles*' appendages (Ingham et al., 2020), were also found to be overexpressed.

## 2 MATERIALS AND METHODS

### 2.1 Mosquito populations

Approval for fieldwork was granted by the National Ethical Committee of Research for Human Health, Cameroon (Authorization Number 2019/06/1167/CE/CNERSH/SP). Gounougou (9°03′00″ N, 13°43′59″ E) is a Guinea savannah village at Lagdo, located in the northern region of Cameroon (Fadel et al., 2019). Mosquito collection was carried out over 4 days in August 2019, with consent from the respective household members. This collection was made 24 months after a previous collection in 2017 (Fadel et al., 2019). Blood‐fed female *Anopheles* mosquitoes, resting indoors, were captured between 6:00 AM and 8:00 AM using a portable electrical aspirator (John W. Hock, Gainesville, FL, USA). Mosquitoes were transferred into paper cups and transported in cooling boxes to the Center for Research in Infectious Disease (CRID), Yaoundé, Cameroon. They were maintained on 10% sucrose, at 25°C ± 2 and 70%–75% relative humidity, until fully gravid. Females were forced to lay eggs in 1.5 mL Eppendorf tubes as previously described (Morgan et al., 2010). Egg batches were transferred into individual paper cups containing clean water for hatching. Following PCR‐based species identification of the F_0_ parents, batches of larvae belonging to the same species were pooled in plastic bowls with water and fed with Tetramin™ baby fish food. The F_1_ female mosquitoes were pooled randomly in cages for subsequent insecticide bioassays and molecular analyses.

### 2.2 Molecular identification of *Anopheles* to species level

Following morphological identification of the F_0_ female *Anopheles gambiae* s.l. (Gillies & Coetzee, 1987; Gillies & De Meillon, 1968) that laid eggs, genomic DNA was extracted from head/thorax using Livak's protocol (Livak, 1984). Identification to the species level was carried out using the SINE200 PCR assay for the *An*. *gambiae* complex (Santolamazza et al., 2008).

### 2.3 Evaluation of sporozoite infection rate

The F_0_ *An. gambiae* s.l. that laid eggs were assayed to identify *Plasmodium* infection using a TaqMan assay (Bass et al., 2008; Fadel et al., 2019). A nested PCR assay (Snounou et al., 1993) was also conducted on all TaqMan‐positive samples, to validate the results.

### 2.4 WHO insecticide susceptibility bioassays

Susceptibility tests were conducted according to the WHO protocol (WHO, 2016) with discriminating concentrations of deltamethrin (0.05%), permethrin (0.75%), DDT (4%), bendiocarb (0.1%), and malathion (5%; all insecticide‐treated papers purchased from the University of Sains Malaysia, reference: WHO/VBC/81.806). Potency of the papers was verified by conducting bioassays with the susceptible Ngousso laboratory colony, originally from Cameroon (Mitchell et al., 2012). Three to four replicates of 20–25 F_1_ females (2–4 days old, unfed) were exposed to each insecticide for 1 h. Following exposure, mosquitoes were transferred to holding tubes and supplied with 10% sucrose. Mortality was scored 24 h post‐exposure. Two replicates of 22 and 24 unexposed females each were used as controls.

### 2.5 Test of pyrethroid resistance intensity

To measure pyrethroid resistance intensity, susceptibility tests were repeated but using 5× and 10× the discriminating concentrations of permethrin (WHO, 2016). Insecticide papers used in this experiment were also purchased from the University of Sains Malaysia. Four replicates each of 20–25 F_1_ females were exposed to 0.75% (1×), 3.75% (5×), and 7.5% (10×) permethrin for 1 h, then transferred to holding tubes and supplied with 10% sucrose. Mortality was scored 24 h post‐exposure. Controls comprised of two replicates of 23 and 21 females exposed to untreated papers.

### 2.6 Synergist bioassays with PBO and DEM

To investigate the role of GSTs in pyrethroid/DDT resistance, permethrin and DDT bioassays were carried out after pre‐exposure to 8% DEM. Experiments were conducted using the WHO protocol (WHO, 2016). Seven replicates of 20–22 F_1_ females (*n* = 149) were pre‐exposed to DEM for 1 h and transferred immediately to tubes containing papers impregnated with either 0.75% permethrin (4 tubes) or 4% DDT (3 tubes). After 1 h of exposure, mosquitoes were transferred to holding tubes, supplied with 10% sugar, and mortality was recorded after 24 h. For each insecticide, 25 females exposed to DEM only were used as a control. To investigate the role of cytochrome P450s in pyrethroid resistance, a permethrin synergist bioassay was conducted with PBO. Because of very low mortalities observed with the discriminating concentration of permethrin, four replicates each of 20–24 unfed F_1_ females (2–4 days old) were pre‐exposed to 4% PBO for 1 h followed by exposure to 1×, 5×, and 10× permethrin for 1 h. Mosquitoes were treated as described above and mortality was recorded after 24 h. Two replicates of 22 females each exposed to PBO only were used as a control.

### 2.7 Identification of the molecular mechanisms of pyrethroid resistance using RNA‐seq

#### 2.7.1 RNA extraction, library preparation, and sequencing

RNA was extracted using the Arcturus PicoPure RNA isolation Kit (Applied Biosystems, CA, USA) from three pools of eight F_1_ *An*. *coluzzii* female mosquitoes (2–4 days old) alive after exposure to 1× (0.75%) permethrin (Resistant, R), unexposed F_1_ females (Control, C), and from unexposed mosquitoes of the fully susceptible *An*. *coluzzii* laboratory colony, Ngousso (Susceptible, S). RNA isolation was carried out following the manufacturer's protocol with Dnase I treatment to remove contaminating DNA. The quantity and quality of RNA pools were measured using a NanoDrop spectrophotometer (ThermoFisher, MA, USA) and Bioanalyzer (Agilent, CA, USA).

RNA‐seq library preparation, sequencing, and initial data quality control were carried out by the Center for Genomic Research (CGR), University of Liverpool, UK. RNA samples were subjected to poly(A) mRNA enrichment and dual‐indexed, strand‐specific RNA‐Seq libraries were prepared using the NEBNext poly(A) selection and Ultra Directional RNA library preparation Kits. Libraries were sequenced on a single lane of Illumina HiSeq 4000 (paired‐end, 2 × 150 bp sequencing). Quality control and post processing of read data, including removal of low‐quality bases and sequencing adapters, were carried out by CGR. Briefly, the raw data fastq files were trimmed of Illumina adapter sequences using Cutadapt version 1.2.1 (Martin, 2011) with option ‐O 3 so that the 3′ end of any reads that matched the adapter sequence for 3 bp or more were trimmed. Low basecall‐quality regions of reads were removed using Sickle version 1.200 (Joshi & Fass, 2011) with a minimum window quality score of 20. Reads shorter than 20 bp after trimming were removed. Read statistics were generated using fastq‐stats from EAUtils (Aronesty, 2011).

#### 2.7.2 Differential gene expression analysis

Paired reads for each replicate were aligned to the *An*. *gambiae* reference transcriptome AgamP4.10 (archived at https://www.vertorbase.org/) in salmon (0.11.4), using “validate mappings,” “seqBias,” “gcBias,” and “rangeFactorizationBins 4” flags. Salmon results were converted into a gene expression matrix using the Bioconductor package “tximport” for input to DESeq2 (1.26.0, script file). For comparison of field‐collected exposed mosquitoes versus unexposed mosquitoes, versus susceptible laboratory colony mosquitoes, an absolute threshold of fold change (FC) >2 was used to define differentially expressed genes with the false discovery rate adjusted *p*‐values of 0.05 applied for significance (R code, File S1).

For the differentially expressed genes of all comparisons, a topGO gene enrichment analysis against *An. gambiae* (AgamP1.10) was performed and transcript functional annotation was conducted using EggNOG (Huerta‐Cepas et al., 2019).

#### 2.7.3 Detection of selective sweeps

To detect signatures of positive directional selection (selective sweeps) in the metabolic resistance genes of interest, a Snakemake RNA‐seq population genetics pipeline RNA‐seq‐Pop was utilized (Ibrahim et al., 2023; Nagi et al., 2022). The pipeline aligns RNA‐seq reads to the reference genome, then calls genomic variants with Freebayes, at a user‐provided level of ploidy (in this case, 16; from 8 diploid mosquitoes in each pool). Then, per‐gene Tajima's D (Tajima, 1989) for each population and *F*st (Hudson et al., 1992) between population pairs are estimated. This was performed against all SNPs passing quality missingness filters. Hudson's *F*st scans were run taking the average of protein‐coding gene, as opposed to in windows. Population genetics statistical analyses were calculated in scikit‐allel v1.2.1 (Miles et al., 2020).

#### 2.7.4 Detection of chromosomal inversion polymorphisms and metabolic resistance genes within its breakpoints

A modified version of the Python3 program, compkaryo (Love et al., 2019), was used to karyotype the major phenotypically important *An*. *coluzzii*/*gambiae* inversion polymorphisms on chromosome 2, and calculate their frequencies, using previously identified single‐nucleotide polymorphism (SNP) variants associated with each inversion orientation. This allows high confidence prediction of genotypes of the six common polymorphic inversions on chromosome 2, in the sequenced field *An. coluzzii*, as well as in Ngousso. Compkaryo uses the Ag1000 database (The Anopheles gambiae 1000 Genome Consortium, 2017) by leveraging a subset of cytologically karyotyped specimens to computationally karyotype whole genome sequence data. Modification in the Snakemake pipeline allowed for variable ploidy (useful in the case of replicates from our pooled RNA sequencing samples).

### 2.8 qRT‐PCR transcriptional profiling of metabolic resistance genes

The expression profile of a panel of upregulated genes from RNA‐seq (plus other candidate genes previously linked to resistance) was validated using qRT‐PCR. These genes include six cytochrome P450s: *CYP6P3* (Müller et al., 2008), *CYP6M2* (Edi et al., 2014; Stevenson et al., 2011), *CYP6Z1* (Chiu et al., 2008), *CYP6Z2* (Ibrahim et al., 2023; McLaughlin et al., 2008), *CYP4G16* (Balabanidou et al., 2016), and *CYP4G17* (Kefi et al., 2019); the glutathione *S*‐transferase gene *GSTe2* (Ibrahim et al., 2023; Mitchell et al., 2014); and three SAP genes, *SAP1*, *SAP2* (Ingham et al., 2020), and *SAP3*. Total RNA was extracted from three pools each of 10 F_1_ unexposed, 10 F_1_ females alive from 1×‐, 5×‐, and 10× permethrin‐alive mosquitoes, and from the Ngousso colony. Extractions were conducted using the PicoPure RNA Isolation Kit (Life Technology, CA, USA). Total RNA (1 μg each) from three biological replicates of 1×‐, 5×‐, and 10× permethrin‐alive females, unexposed females, and Ngousso females, respectively, was used as template for cDNA synthesis using superscript III (Invitrogen, Carlsbad, CA, USA) with Oligo‐dT20 and RNase H (New England Biolabs, Massachusetts, USA). Serial dilutions of cDNA were used for each gene to establish standard curves, to assess PCR efficiency and quantitative differences between samples. Amplification was carried out using a MX Brilliant III Ultra‐Fast SYBR QPCR Master mix (Agilent, Santa Clara, CA, USA). 10 ng of cDNA from each sample was used as template in a three‐step program involving a denaturation at 95°C for 3 min, followed by 40 cycles each of 10 s at 95°C and 10 s at 60°C, and a last step of 1 min at 95°C, 30 s at 55°C, and 95°C 30 s (Kwiatkowska et al., 2013). Primers utilized are provided in Table S1. The relative expression levels of and fold change in the candidate gene compared to Ngousso samples were calculated using the 2^−ΔΔCT^ method previously described (Schmittgen & Livak, 2008), after normalization to two housekeeping genes, the ribosomal protein *RPS7* (AGAP010592), and an elongation factor (AGAP005128).

### 2.9 Genotyping of voltage‐gated sodium channel (VGSC) gene knockdown resistance mutations

To assess the role of the 1014F, 1014S, and 1575Y knockdown resistance mutations in pyrethroid/DDT resistance, their frequencies were first established in F_0_ females using TaqMan assays, as previously described (Bass et al., 2007; Ibrahim, Fadel, Tchouakui, et al., 2019; Jones et al., 2012). Forty two genomic DNA samples extracted from *An*. *coluzzii* collected in Gounougou in 2017 and previously genotyped for 1014F (Fadel et al., 2019) were used for comparative genotyping of the 1014S *kdr* mutation, and 71 *An*. *coluzzii* collected in 2017 were used to genotype the 1575Y *kdr* mutation. The 1014F *kdr* genotyping data obtained in 2017 (Fadel et al., 2019) were used for comparison with data from 2019. For the detection of the 1575Y *kdr* mutation, primer/probe (1575 Forward: TGGACTGCTAGAAATGTTCATGACA, 3′‐NFQ 3′‐TTTTTCATTGCATAATAGTAC 6‐FAM and 1575‐Reverse: CGAGGAATTGCCTTTAGAGGTTTCT, 3′‐NFQ ATTTTTTTCATTGCATTATAGTAC HEX) was used. A set of three positive samples of known genotypes including homozygous‐resistant, heterozygous‐resistant, and homozygous‐susceptible for 1014F, 1014S, and 1575Y was used as positive controls for each of the three mutations.

To investigate the frequency of 1014F *kdr* mutation in the highly resistant mosquitoes, F_1_ females exposed to 5× and 10× permethrin were also genotyped. DNA for genotyping was extracted from 23 females alive after exposure to 1× permethrin, 21 survivors of 5× permethrin, and 24 survivors of 10× permethrin.

### 2.10 Genotyping of the G119S *acetylcholinesterase‐1* mutation

To investigate the presence of a carbamate and organophosphate resistance‐conferring *ace*‐1 mutation (Weill et al., 2004), TaqMan genotyping of the 119S mutation was carried out using 67 F_0_ females randomly selected from the 2019 field collection. In addition, seven F_1_ females that survived bendiocarb exposure and nine dead females were also screened. Genotyping was done using the TaqMan assay, as previously described (Bass et al., 2010; Ibrahim, Fadel, Tchouakui, et al., 2019). Three positive samples of known genotypes: homozygous‐resistant for the 119S *ace*‐1 mutation, heterozygous‐resistant for 119S, and homozygous‐susceptible for G119 were run for comparison, in addition to a negative control made up of 1 μL ddH_2_O.

### 2.11 Data analysis

Sporozoite rate was calculated as the percentage of female mosquitoes with sporozoites relative to the total number of mosquitoes tested. Mortality rates for each insecticide were defined using WHO guidelines: resistant mosquitoes (<90% mortality), potentially resistant (mortality of 90%–98%), and susceptible (mortality >98%). Results of mortalities from synergist bioassays were compared to the values obtained from conventional bioassays (insecticides alone) using a two‐tailed chi‐squared test of independence in GraphPad Prism 7.02 (GraphPad Inc., La Jolla, CA, USA). The *kdr* mutation frequencies were calculated by type, locus, allele, and year for analysis.

RNA‐Seq‐pop uses Kallisto to quantify reads and the R packages DESEq2 for differential expression testing. Volcano plots were created in ggplot2 (Wickham et al., 2016) to evaluate the relationship between differentially expressed genes (level of significance versus log_2_FC for each gene) and plot the expression of significant genes using heatmaps (pHeatmap package) (Kolde, 2019). Principal component analyses were performed with R FactoMineR and Factoshiny packages (Lê et al., 2008; Vaissie et al., 2020). Venn diagrams were created with the R Venn diagram package to summarize the numbers of differentially expressed genes between resistant field‐mosquitoes, unexposed field‐mosquitoes, and susceptible colony mosquitoes (Chen, 2018). Differences in gene expression from qRT‐PCR were tested using unpaired Student's *t* tests.

## 3 RESULTS

### 3.1 Mosquito species composition and *Plasmodium* infection

Overall, 270 mosquitoes were caught indoors in Gounougou. These comprised 17 *An. funestus*, 12 *An. rufipes*, and 48 *Culex* spp. A total of 193 blood‐fed *An*. *gambiae* s.l. females were caught also, of which 188 laid eggs successfully. All female *An. gambiae* s.l. that laid eggs were identified as *An*. *coluzzii*. A total of 101 (52.3%) of the egg batches hatched successfully. Out of the 77 F_0_ females screened for the presence of malaria parasites, only three (3.89%) were infected with *P*. *falciparum*.

### 3.2 Susceptibility bioassays with 1× discriminating concentrations of insecticides

**FIGURE 1:**
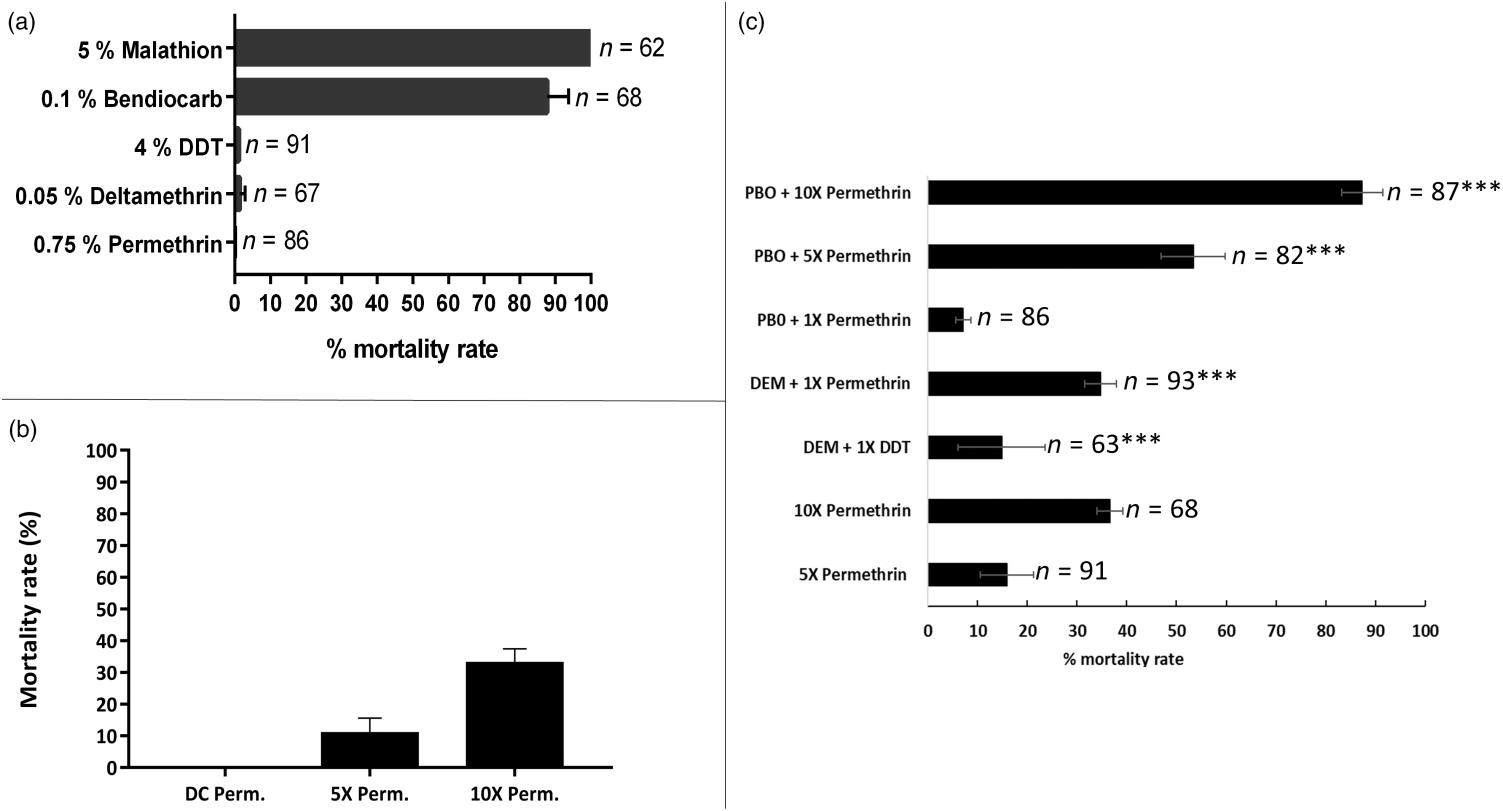
Resistance profiles of *An. coluzzii* from Gounougou. (a) Results of susceptibility tests with several insecticides from different classes. Error bars represent standard error of the mean. *n*, total number of mosquitoes tested; (b) Determination of resistance intensity with 5× and 10× discriminating concentrations of permethrin. Results are the average of percentage mortalities from four replicates of each in female F_1_ *An. coluzzii* populations. DC, discriminating concentration; Perm., permethrin; (c) Effect of pre‐exposure to synergist DEM and PBO against DDT and permethrin in female F_1_ *An. coluzzii* populations, respectively. ***Statistically significant at *p* < 0.001, in chi‐square test of independence between results from synergists bioassays and standard.

High pyrethroid resistance was obtained from Gounougou *An. coluzzii*, with no mortality at all from permethrin (Figure 1a, File S2) and a low mortality of only 1.45% ± 1.45 from deltamethrin. Exceptionally low mortality (1.14% ± 1.14) was also observed from DDT exposure. Moderate resistance to bendiocarb was observed, with mortality of 87.78% ± 5.94, and full susceptibility to malathion, with 100% mortality. All *An*. *coluzzii* Ngousso strain mosquitoes exposed to these five insecticides were fully susceptible.

### 3.3 Measurement of pyrethroid resistance intensity

To measure the intensity of pyrethroid resistance, susceptibility tests were performed with 5× and 10× the discriminating concentrations of permethrin. Bioassays revealed a high intensity of resistance, with mortality increasing from 0% for 1× permethrin to only 10.95 ± 4.68% for 5× permethrin (*χ* ^2^ = 9.904, *df* = 1, *p* = 0.0016; Figure 1b) and only 33.07 ± 4.37% for 10× permethrin (*χ* ^2^ = 34.32, *df* = 1, *p* < 0.0001). Extended raw data have been provided in the supplementary material (File S2).

### 3.4 Investigation of the role of major detoxification gene families using synergist‐insecticide bioassays

Pre‐exposure to DEM recovered some susceptibility to permethrin and DDT, with mortalities increasing from 0% in conventional bioassay with 1× permethrin to 34.75 ± 3.23% (*χ* ^2^ = 33.87, *df* = 1, *p* = 0.0001) in synergized bioassay, and from 1.14 ± 1.14% for 1× DDT to 14.88 ± 8.74% (*χ* ^2^ = 37.41, *df* = 1, *p* = 0.0001) in synergized bioassay (Figure 1c). These findings suggest the potential role of glutathione *S*‐transferases in DDT and permethrin resistance.

Significant recovery of permethrin susceptibility was observed with 5× and 10× permethrin after PBO pre‐exposure, with a mortality of rate of 53.39 ± 6.44%, compared to 15.96 ± 5.36% mortality with no PBO (*χ* ^2^ = 28.35, *df* = 1, *p* = 0.0001) and 87.30 ± 4.10% compared to 36.59 ± 2.57% without PBO (*χ* ^2^ = 43.03, *df* = 1, *p* = 0.0001), respectively. These findings suggest that cytochrome P450s play a role in permethrin resistance, with parallel, alternative non‐P450 mechanism also contributing to resistance. Extended raw data have been provided in the supplementary (File S2).

### 3.5 Differential gene expression and gene ontology (GO) analysis

**FIGURE 2:**
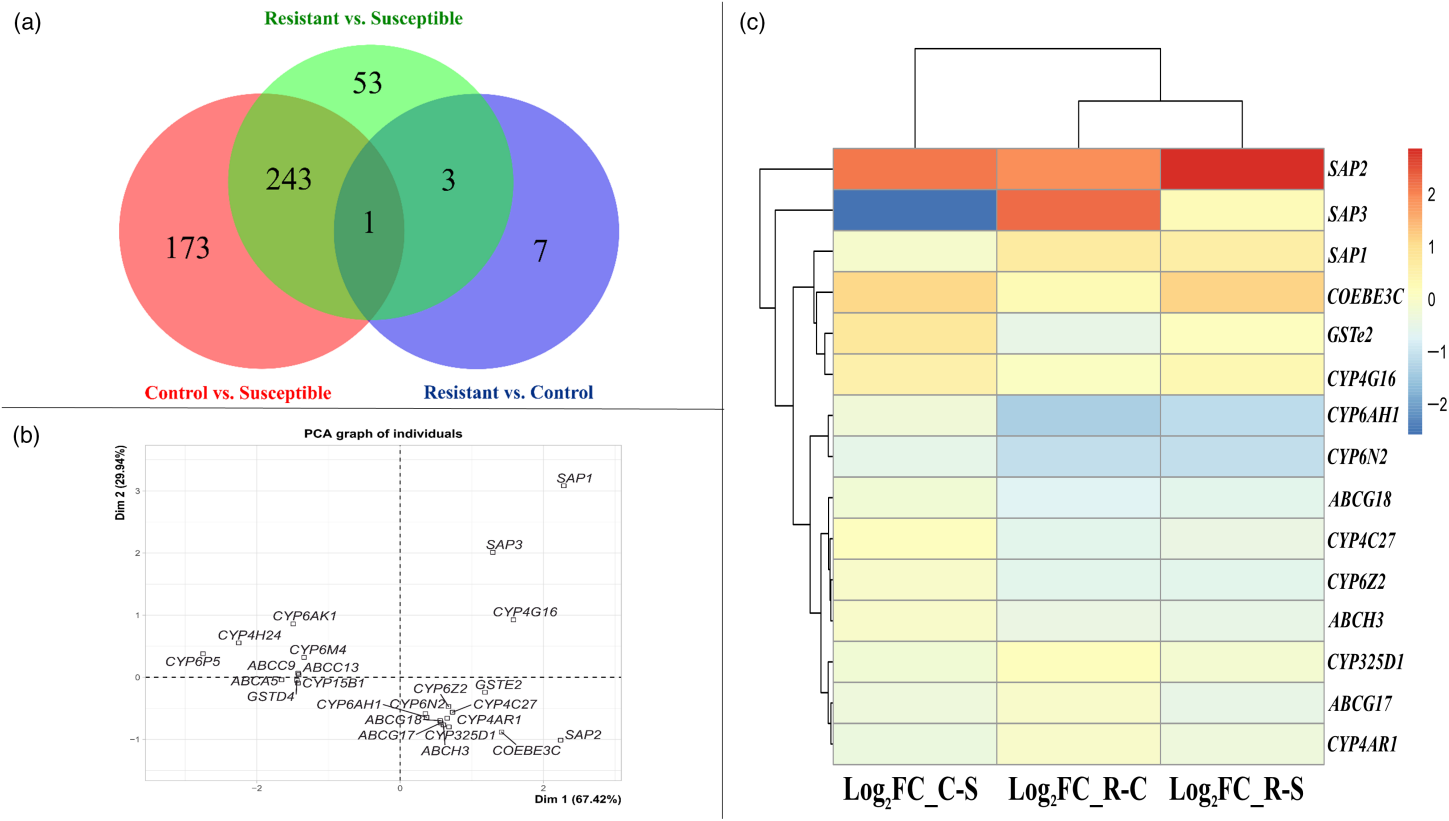
Differential gene expression analysis. (a) Venn diagram showing the number of common and specific differentially expressed genes for each comparison between Gounougou and Ngousso. (b) Principal component analysis (PCA) plot showing the most individuals contributing resistant genes involved in the plan of construction. The labelled individuals are those with higher contribution to the plane construction. (c) pHeatmap showing relative expression of the potential first 15 detoxification genes differentially expressed between different comparisons qt *p*‐value < 0.05 and log_2_FC > 2.

The number of read pairs mapped to the *An. gambiae* reference transcriptome *Agam* P4.10 after filtering ranged from 19,723,450 (98.42%) to 32,054,416 (98.42%). A table of raw read counts per gene for each replicate is given in Table S2. Principal component analysis broadly showed that replicates clustered within treatments which clustered separately from one another (Figure S1a). Comparing Gounougou mosquitoes surviving permethrin exposure to Ngousso susceptible mosquitoes showed strikingly different transcriptomic profiles. The number of significantly differentially expressed genes for resistant versus susceptible (R‐S) was 407 (300 up‐ and 107 down‐regulated in R) (Figure S1b (A)) and for control versus susceptible (C‐S) it was 556 (417 up‐ and 139 down‐regulated in C) (Figure S1b (B)). For resistant versus control (R‐C), there were 25 differentially expressed genes (11 up‐ and 14 down‐regulated in R; Figure S1b (C)), increasing to 103 (41 up‐ and 62 downregulated in R) at a lower FC threshold (1.5 rather than 2). On comparing genes commonly overexpressed in (R‐C)/(R‐S)/(C‐S), only one unknown gene (*AGAP001983*) was commonly overexpressed in all three comparisons (Figure 2a). A total of 244 genes had shared differential expression in the R‐S and C‐S comparisons.

The 50 genes most differentially expressed in common for R‐S and C‐S comparisons are given in Table S3. These included the highly upregulated genes; *cubilin* (AGAP005526); mitochondrial ribosomal protein (*mRps24*, AGAP012833); diverse CLIP‐domain serine proteases, for example, *CLIPB12* (AGAP009217), *CLIPB18* (AGAP009215), and *CLIPE1* (AGAP008091); unknown sulfotransferase (AGAP012696); Toll protein (AGAP001002); D‐amino acid oxidase (AGAP001116); eukaryotic large subunit ribosomal RNA (AGAP028931); several leucine‐rich immune proteins, for example, *LRIM12* (AGAP005496), *LRIM16A* (AGAP028028), and *LRIM16A* (AGAP028064); and a cuticular protein (*CPR131*, AGAP010123). Other genes including UDP‐N‐acetyl‐alpha‐D‐galactosamine (AGAP010367), V‐type proton ATPase subunit a (AGAP012818), prophenoloxidase (*PPO9*, AGAP004978), Kelch‐like protein 28 (AGAP012550), carbonic anhydrase I (AGAP013402), and thioester‐containing protein (*TEP8*, AGAP010831) were also upregulated.

Further attention was given to genes known to be involved in metabolic insecticide resistance in *Anopheles* and/or other insects. These included cytochrome P450s, glutathione *S*‐transferases, carboxylesterases, ABC‐transporters, cuticular proteins, chemosensory proteins, and SAPs, as well as chymotrypsins and aquaporins. Analysis of the data from the list of genes significantly differentially expressed (FDR‐adjusted *p* < 0.05 and FC > 2) revealed the major, upregulated metabolic resistance genes in Gounougou for R‐S and C‐S comparisons. The top 50 genes, with FC > 2 for either R‐S or C‐S, or FC > 2, for both comparisons are presented in Table 1.

**TABLE 1:** Common metabolic detoxification genes differentially and constitutively upregulated in Gounougou *An. coluzzii* at FDR‐adjusted *p* < 0.05.

Gene ID	R‐S	C‐S	Gene description
AGAP028491	1.42	2.04	Aquaporin, AQP2
AGAP010326	2.35	3.52	Aquaporin, AQP3
AGAP002391	1.56	2.21	Carboxylesterase, COEAE5O
AGAP005372	3.47	3.15	Carboxylesterase, COEBE3C
AGAP005837	1.52	2.03	Carboxylesterase, COEJHE5E
AGAP006191	5.62	5.04	Chitinase, Cht24
AGAP013260	1.46	2.03	Chitinase, Cht5‐5
AGAP006898	3.14	2.83	Chitinase, Cht6
AGAP006709	4.17	6.47	Chymotrypsin‐2, CHYM1
AGAP006710	2.69	4.80	Chymotrypsin‐2, CHYM2
AGAP006711	4.11	6.70	Chymotrypsin‐2, CHYM3
AGAP000989	2.22	1.66	Cuticular protein, CPAP3‐A1a
AGAP000988	1.84	2.49	Cuticular protein, CPAP3‐A1c
AGAP000986	2.37	2.60	Cuticular protein, CPAP3‐D
AGAP004690	2.91	1.97	Cuticular protein, CPF3
AGAP009759	2.66	3.31	Cuticular protein, CPLCP12
AGAP010887	1.54	2.14	Cuticular protein, CPR113
AGAP003375	3.40	3.93	Cuticular protein, CPR114
AGAP000820	2.38	2.52	Cuticular protein, CPR125
AGAP010123	6.39	6.92	Cuticular protein, CPR131
AGAP006868	2.07	1.68	Cuticular protein, CPR140
AGAP007042	1.92	2.68	Cuticular protein, CPR62
AGAP009868	4.47	6.11	Cuticular protein, CPR73
AGAP009871	1.54	2.30	Cuticular protein, CPR75
AGAP009874	1.69	3.03	Cuticular protein, CPR76
AGAP002204	1.95	2.25	Cytochrome P450, CYP325D1
AGAP002417	1.81	2.16	Cytochrome P450, CYP4AR1
AGAP009246	1.66	2.48	Cytochrome P450, CYP4C24
AGAP001076	2.53	2.73	Cytochrome P450, CYP4G16
AGAP007480	0.86	2.24	Cytochrome P450, CYP6AH1
AGAP008206	0.92	2.05	Cytochrome P450, CYP6N2
AGAP008218	1.48	2.39	Cytochrome P450, CYP6Z2
AGAP009194	2.32	2.93	Glutathione S‐transferase, GSTe2
AGAP011216	3.77	0.16	Heme peroxidase, HPX16
AGAP000051	2.19	1.24	Heme peroxidase, HPX5
AGAP002353	2.94	3.89	Lipase
AGAP000184	3.08	3.70	Malate dehydrogenase
AGAP008051	2.86	2.33	Sensory appendage protein 1, SAP1
AGAP008052	5.47	3.88	Sensory appendage protein 2, SAP2
AGAP008054	2.40	0.64	Sensory appendage protein 3, SAP3
AGAP013327	1.12	2.81	Sulfotransferase, HPX15
AGAP008654	1.87	2.53	Thioester‐containing protein 12, TEP12
AGAP008368	1.59	2.24	Thioester‐containing protein 14, TEP14
AGAP010812	1.53	2.72	Thioester‐containing protein, TEP4
AGAP010831	6.03	3.93	Thioester‐containing protein, TEP8
AGAP010815	2.26	2.88	Thioesther‐containing protein, TEP1
AGAP009468	1.61	2.17	Transporter, ABCG17
AGAP028729	1.46	2.27	Transporter, ABCG18
AGAP002060	1.59	2.36	Transporter, ABCH3
AGAP012818	3.96	4.23	V‐type proton ATPase subunit a

Of these, 23 genes were commonly overexpressed between the R‐S and C‐S comparisons, including six cuticular proteins: *CPR131* (AGAP010123), *CPR73* (AGAP009868), *CPR114* (AGAP003375), *CPR125* (AGAP000820), *CPAP3‐D* (AGAP000986), and *CPLCP12* (AGAP009759), with fold changes for *CPR131* in R‐S and C‐S comparisons of 6.39 and 6.92; for *CPR73*, 4.47 and 6.11; for *CPR114*, 3.40 and 3.93; for *CPR125*, 2.38 and 2.52; for *CPAP3‐D*, 2.37 and 2.60; and for *CPLCP12*, 2.66 and 3.31, respectively; two chitinases: *Cht24* (AGAP006191) and *Cht6* (AGAP006898), with FC for *Cht24* in R‐S and C‐S comparisons of 5.62 and 5.04, and for *Cht6*, 3.14 and 2.83, respectively; three chymotrysins: *CHYM1*/AGAP006709, *CHYM2*/AGAP006710, and *CHYM3*/AGAP006711, with FC for *CHYM1* in R‐S and C‐S of 4.17 and 6.47; for *CHYM3*, 4.11 and 6.70; and for *CHYM2*, 2.69 and 4.80, respectively; two SAPs: *SAP2* (AGAP008052) and *SAP1* (AGAP008051), with FC for *SAP2* in R‐S and C‐S comparisons of 5.47 and 3.88, and for *SAP1*, 2.86 and 2.33, respectively; two thioester‐containing proteins: *TEP8* (AGAP010831) and *TEP1* (AGAP010815), with FC for *TEP8* in R‐S and C‐S comparisons of 6.03 and 3.93, and for *TEP1*, 2.26 and 2.88, respectively. Other genes upregulated in R‐S and C‐S comparisons included a carboxylesterase, *COEBE3C* (AGAP005372), with FC of 3.47 and 3.15, respectively for R‐S and C‐S comparisons; a V‐type proton ATPase subunit a (AGAP012818), with FC of 3.96 and 4.23; a malate dehydrogenase (AGAP000184), with FC of 3.08 and 3.70; a lipase (AGAP002353), with FC of 2.94 and 3.89; an aquaporin, *AQP3* (AGAP010326), with FC of 2.35 and 3.52; a P450, *CYP4G16* (AGAP001076), with FC of 2.53 and 2.73 in R‐S and C‐S; and a glutathione *S*‐transferase, *GSTe2* (AGAP009194), with FC in R‐S and C‐S of 2.32 and 2.93, respectively.

For the R‐S comparison only, other genes upregulated included heme peroxidases *HPX16* (AGAP011216, FC = 3.77) and *HPX5* (AGAP000051, FC = 2.19); cuticular proteins, *CPF3* (AGAP004690, FC = 2.91), *CPAP3‐A1a* (AGAP000989, FC = 2.22), *CPR140* (AGAP006868, FC = 2.07); and *SAP3* (AGAP008054, FC = 2.40). For the C‐S comparison only, other upregulated genes included cuticular proteins *CPR76* (AGAP009874, FC = 3.03), *CPAP3‐A1c* (AGAP000988, FC = 2.49), *CPR62* (AGAP007042, FC = 2.48), and *CPR75* (AGAP009871, FC = 2.30); thioester‐containing proteins *TEP4* (AGAP010812, FC = 2.72) and *TEP12* (AGAP008654, FC = 2.53); and a heme peroxidase, *HPX15* (AGAP013327, FC = 2.81). The potential detoxification genes differentially expressed between different comparisons from this list (Table 1) are visualized as principal components plot and heatmap in Figure 2b,c, respectively.

Several genes were downregulated for R‐S and C‐S comparisons, particularly the cytochrome P450s, ATP‐binding cassette transporters, and cuticular protein families. The most common detoxification and metabolic genes differentially and constitutively downregulated at FDR adjusted *p*‐value < 0.05 are listed in Table S4. Among the cytochrome P450s, *CYP6P5* (AGAP002866), *CYP4H24* (AGAP013490), and *CYP6AK1* (AGAP010961) were the most downregulated. For the C‐S comparison only, two transporter genes, *ABCC13* (AGAP009799) and *ABCCS* (AGAP012156), were the most downregulated. *TRYP6* (AGAP008290), *TRYP7* (AGAP008283), and *TEP6* (AGAP010814) were the most common other detoxification genes significantly downregulated.

### 3.6 Validation of transcriptional profile of resistance genes using qRT‐PCR

**FIGURE 3:**
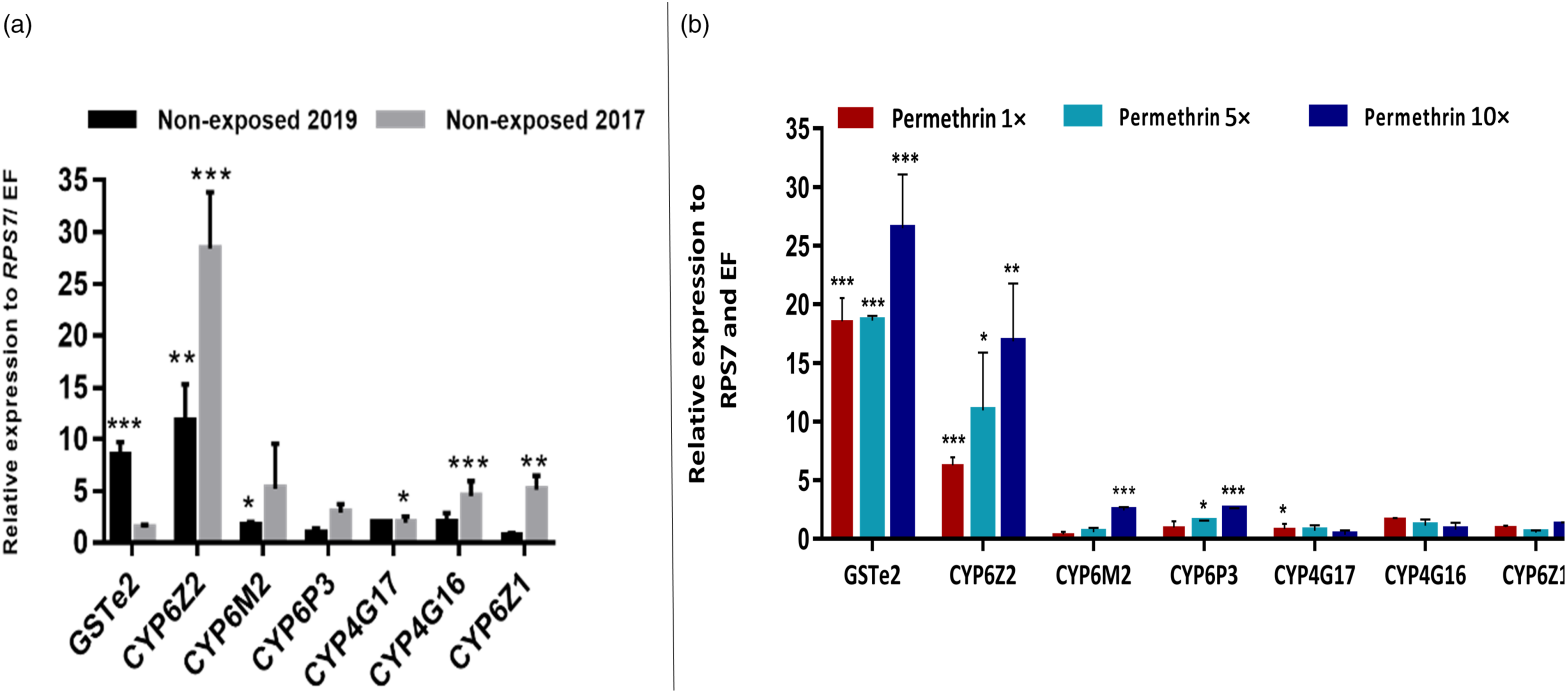
Transcription profile by qRT‐PCR of resistance genes in Gounougou *An. coluzzii* comparing their patterns of expression between (a) nonexposed and (b) DC permethrin‐, 5× permethrin‐, and 10× permethrin‐resistant mosquitoes with susceptible Ngousso strain. DC, discriminating concentration. Error bars represent standard error of the mean. *p < 0.01, **p < 0.001; ***p < 0.0001.

The qRT‐PCR revealed seven out of the ten genes screened to be overexpressed in unexposed females compared with Ngousso females (Figure 3a), with the highest expression among unexposed seen for the cytochrome P450, *CYP6Z2* for both collections from 2017 and 2019. The level of expression of *CYP6Z2* in unexposed females collected in 2019 had significantly decreased 2.4 times, compared to the fold change obtained in samples from 2017 (FC: 28.4× vs. 11.7×, *p* < 0.0177). In contrast, the overexpression of *GSTe2* had significantly increased five times in the 2019 collection (fold change: 8.4× vs. fold change: 1.4×, *p* < 0.0043) compared to 2017 (Figure 3a).

*GSTe2* is the most consistently overexpressed metabolic gene with a fold change of 18.5× [95% (*CI* 8.6–12.0), *p* < 0.0001], 18.6× [95% (*CI* 9.8–10.8), *p* < 0.0001], and 26.5× [95% (*CI* 11.1–18.8), *p* < 0.0004], in the females exposed to 1×, 5×, and 10× permethrin collected in 2019, compared with Ngousso (Figure 3b). Significant differences were observed when comparing the fold changes of 1× permethrin to 10×permethrin, for *GSTe2* [95% (*CI* = 0.5–8.8), *p* < 0.03] (Figure 3b), and 5× permethrin resistant versus 10× permethrin resistant [95% (*CI* = 0.8–8.4), *p* < 0.02] (Figure 3b). *CYP6Z2* was the second most overexpressed gene with FCs, respectively, of 6.2× [95% (*CI* = 2.8–4.1), *p* < 0.0001], 10.9× [95% (*CI* = 1.9–11.2), *p* < 0.01], 16.8× [95% (*CI* = 5.8–15.1), *p* < 0.003], and 11.7× [95% (*CI* = 3.7–10.4), *p* < 0.004] for 1× permethrin alive, 5× permethrin alive, 10× permethrin alive, and control nonexposed mosquitoes collected in 2019 compared to Ngousso (Figure 3a,b). Significantly higher expression of *CYP6Z2* was observed in 10× permethrin‐resistant [95% (*CI* = 2.3–11.6), *p* < 0.01] and nonexposed females [95% (*CI* = 0.18–7.02), *p* < 0.04] compared to values obtained from 1× permethrin‐resistant females.

*CYP6P3* was also significantly upregulated in 10× permethrin‐resistant females (FC = 2.5) compared to Ngousso [95% (*CI* = 2.4–4.1), *p* < 0.0004]. This difference in expression was moderate for *CYP6P3* in 5× permethrin‐resistant samples with FC 1.5× higher than susceptible mosquitoes [95% (*CI* = 0.5–2.1), *p* < 0.01]. *CYP6M2* was significantly upregulated in nonexposed females collected in 2017 with a fold 5.2× higher than Ngousso (*p* < 0.0114) strain (Figure 3a). *CYP6M2* was also significantly overexpressed but only in 10× permethrin‐resistant females collected in 2019, with a FC of 2.4× compared to Ngousso [95% (*CI* = 5.5–10.11), *p* < 0.0007] (Figure 3b). *CYP4G16* and *CYP6Z1* were significantly upregulated with a fold change of 4.4× (*p* < 0.0005) and 5.1× (*p* < 0.0038) only in nonexposed females collected in 2017 compared to Ngousso (Figure 3a). *CYP4G17* was also significantly more overexpressed though at moderate levels, with a fold change of 1.7 × [95% (*CI* = 2.9–18.7), *p* < 0.0188] and 1.8× [95% (*CI* = 1.7–17.1), *p* < 0.0276], respectively, in 1× permethrin‐resistant females and nonexposed compared to Ngousso (Figure 3a,b).

Transcriptional analysis of the candidate *SAP2*, known to confer pyrethroid resistance in *An. gambiae* s.l., revealed that contrary to RNA‐seq findings, it is not overexpressed in *An*. *coluzzii* from Gounougou, with 0.52‐ and 0.98‐fold change, respectively, in 2017 and 2019. *SAP1* and *SAP3* were also found not to be upregulated, with 0.10‐ and 0.18‐fold change in 2017, and 0.15‐ and 0.44‐fold change in 2019, respectively.

### 3.7 Detection of signatures of selective sweeps

Signatures of selection were investigated in the major metabolic resistance genes, by estimating Tajima's D per gene within populations and *F*st per gene between population pairs. These tests of neutrality revealed several genes exhibiting genetic differentiation or possibly undergoing expansion. Among the overexpressed metabolic genes in Gounougou, six genes were possibly undergoing genetic differentiation (File S3). These include *TEP1* and *TEP3*, with average Tajima's D of −1.88 and −1.06 respectively, in Gounougou, and *F*st of 3 L chromosomal, which was calculated as 0.52 and 0.58 respectively, *CPR15* (average Tajima's D = −0.80 for Gounougou, and *F*st of 0.29 calculated for the chromosome 2 L), *CPAP3‐A1a* (average Tajima's D = −1.21), *CPAP3‐A1b* (average Tajima's D = −1.57), and *GSTU1* (average Tajima's D = −1.34) with average *F*st of X chromosome, which were calculated as 0.63, 0.52, and 0.29 respectively. Positive selection was also evident in the peptide alpha N‐acetyltransferase (AGAP002284, Tajima's D = −2.07 and *F*st = 0.07). Four GST genes with average *F*st of 3R chromosome, *GSTe1* (AGAP009195, Tajima's D = −1.57, *F*st = 0.03), *GSTe3* (AGAP009197, Tajima's D = −1.83, *F*st = 0.006), *GSTe4* (AGAP009193, Tajima's D = −1.68, *F*st = 0.014), and *GSTe5* (AGAP009192, Tajima's D = −1.64, *F*st = 0.02) were possibly undergoing differentiation as well.

### 3.8 Detection of chromosomal inversion polymorphisms and metabolic resistance genes within its breakpoints

The frequencies of the major inversion polymorphisms in chromosome 2 were calculated, considering ploidy (for each gene up to 16 alleles, from 8 diploid individual female mosquitoes pooled for RNA extraction, for each replicate). Table S5 shows the frequencies of the respective inversions for the population from Gounougou, as well as for Ngousso. High frequency of the 2La, 2Rb, and 2Rc inversions were observed, in contrast to 2Rd, 2Rj, and 2Ru that occurred at lower frequencies. The 2La inversion was found to be fixed in the field population (99.23% in Gounougou), in contrast with Ngousso, in which its frequency was only 6.96%. Similar patterns were observed with the 2Rb inversion, with a high frequency of 62.74% in Gounougou, but a very low frequency of 4.46% in Ngousso. Frequencies of the 2Rc inversion were 63.02% and 9.89% for Gounougou and Ngousso, respectively. Frequencies of the 2Rd, 2Rj, and 2Ru inversions were 4.94%, 16.14%, and 8.89%, respectively, in Gounougou, while the calculated frequencies of the 2Rd, 2Rj, and 2Ru in Ngousso were 1.04%, 53.12%, and 0%, respectively.

### 3.9 Identification of the knockdown mutations in the voltage‐gated sodium channel

TaqMan genotyping of 68 F_0_ females detected the 1014F, 1014S, and 1575Y mutations at frequencies of 50%, 6.61%, and 10.29%, respectively (Table S6). In samples collected in 2019, 1014F was found at a high frequency at 50%, with heterozygote‐resistant mosquitoes predominant at 64.72% (44/68), and both homozygote resistant and susceptible at equal frequencies of 17.64% (12/68). In comparison, 1014F *kdr* frequency was 65.25% in 2017 samples with heterozygote‐resistant mosquitoes marginally predominant at 45.76% (27/59), homozygote resistant at 42.37% (25/59), and susceptible at 11.87 (7/59).

In contrast, the 1014S mutation was detected at a low frequency of 6.61% in 2019, with homozygote susceptible allele at 86.77% (59/68), and only 13.33% (9/68) were heterozygote resistant. The frequency of the 1014S (6.61%) mutation has decreased two times compared to the frequencies established in 2017 (15.47%). The 1575Y was also present at low frequency of 10.29%, with only one homozygote‐resistant mosquito (1.48%), twelve heterozygote individuals (17.64%), and predominant homozygote susceptible individuals at frequency of 80.88% (55/58). The frequency of the 1575Y (10.29%) mutation has decreased ~5 times compared to the frequencies established in 2017 (51.4%). Also, 10 females were found carrying both 1014F and 1575Y *kdr* mutations in both collections from 2017 and 2019, combined.

**FIGURE 4:**
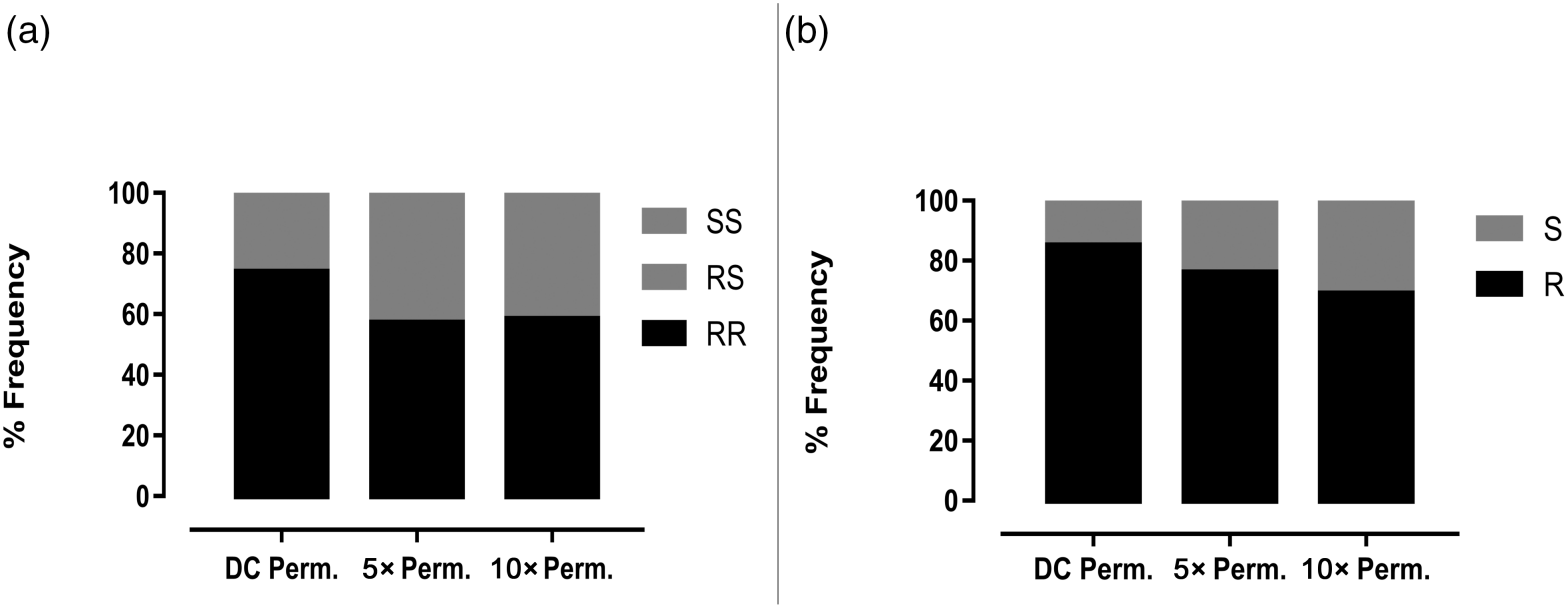
Distribution of genotypes (a) and allele frequencies (b) of the 1014F *kdr* mutation in *An. coluzzii* from Gounougou. Results are relative proportion of each genotype and allele frequencies. R, resistant allele; RR, homozygote‐resistant genotype; RS, heterozygote‐resistant genotype; S, susceptible allele; SS, homozygote‐susceptible genotype.

The distribution of relative proportion of each genotype and allele frequencies of the 1014F *kdr* mutation in F_1_ females which survived 1×, 5× and 10× of the discriminating concentration of permethrin is shown in Figure 4a,b. The 1× discriminating concentration of permethrin had a higher proportion of homozygote‐resistant individuals. All three batches of survivors at 1×, 5×, and 10× exposure of permethrin had high frequencies of the 1014F mutation; however, contrary to expectation, if *kdr* was driving resistance escalation, the frequency of 1014F rather decreased from 1× alive (84.78%), 5× (76.19%) to 10× (68.65%; Figure 4b). Homozygote‐resistant and heterozygote‐ and homozygote‐susceptible individuals were obtained at frequencies of 73.91% (17/23), 21.74% (5/23), and 4.35% (1/23), respectively, for 1× permethrin; 57.14% (12/21), 38.09% (8/21), and 4.47% (1/21), respectively, for 5× permethrin, and 58.34% (14/24), 20.83% (5/24), and 20.83% (5/24), respectively, for 10× permethrin.

### 3.10 Absence of carbamate resistance‐associated mutation G119S in the *ace‐1* gene

The G119S *ace*‐1 mutation known to confer resistance to carbamate and organophosphate insecticides was not detected in any of the seven bendiocarb‐alive and nine bendiocarb‐dead females. Also, the mutation was not detected in any of the 67 field‐collected F_0_ females.

## 4 DISCUSSION

Insecticide resistance escalation is being reported in *Anopheles* mosquitoes across Africa (Ibrahim, Mukhtar, Datti, et al., 2019; Riveron et al., 2019; Riveron, Tchouakui, et al., 2018), and unless urgent action is taken it will reverse the progress made in malaria control using insecticide‐based vector control interventions (Bhatt et al., 2015). To investigate potential insecticide resistance escalation in *An. coluzzii*, the principal malaria vector in northern Cameroon, insecticide resistance profiles were determined and potential molecular mechanisms underlying resistance characterized for a population of *An. coluzzii* from Gounougou in northern Cameroon.

### 4.1 *An. coluzzii* exhibits a low level of *Plasmodium falciparum* infection in Gounougou

*Anopheles coluzzii* was the only member of the *An*. *gambiae* complex collected indoors in this study. This is similar to previous observations from a collection conducted in 2017 in the same locality and in the same month (Fadel et al., 2019), when *An. coluzzii* was the most prevalent species found sympatric with a handful of *An. rufipes* and *An. arabiensis*. The finding of *An. coluzzii* as the only malaria vector in the height of rainy season, coinciding with peak malaria transmission, has been reported elsewhere in Africa, for example, in Sudan/Sahel of northern Nigeria (Ibrahim, Mukhtar, Datti, et al., 2019), in central Sahel of Chad (Ibrahim, Fadel, Tchouakui, et al., 2019), and in Sahel of Niger (Ibrahim, Mukhtar, Irving, et al., 2019). However, this could be due to a bias from indoor collection which could have missed exophilic and zoophilic mosquitoes like *An. arabiensis* (Asale et al., 2017; Duchemin et al., 2001; Russell et al., 2011). *Plasmodium falciparum* remains the only malaria parasite identified in *An*. *coluzzii* from Gounougou, at a slightly lower frequency (not statistically significant) of 3.89% compared to 4.70% in the same species collected in 2017 (Fadel et al., 2019), suggesting ongoing low malaria transmission in the region. These infection rates are higher than 1.26% recorded in 2014 in *An. gambiae* s.l. from North Cameroon (Awono‐Ambene et al., 2018) but lower than that obtained in 2017 in *An. gambiae* from Mibellon, the neighboring region of Adamawa (Menze et al., 2018).

### 4.2 Evidence of escalation of pyrethroid resistance in the Gounougou *An. coluzzii* population

The high DDT resistance observed in the 2019 population was similar to that observed in 2017, while an increase in permethrin resistance was observed in the 2019 collection; with no mortality at all at 1× permethrin compared with 3.75 ± 1.25% in 2017 (Fadel et al., 2019). This increased pyrethroid resistance may be associated with selective pressure acting on an endophilic *An*. *coluzzii* population, imposed by the introduction of pyrethroid insecticides for bed nets in Gounougou since 2006. In addition, based on previous studies in the West and Central Africa, several authors hypothesized that the past and current agricultural use of pyrethroids, DDT, and organophosphates for crop protection leads to the selection of resistant individuals by challenging larval stages with residual insecticide products accumulating in water bodies around cultivated areas (Antonio‐Nkondjio et al., 2008; Dabiré et al., 2009; Yahouedo et al., 2016). Similar trends of increased pyrethroid resistance have been observed also in *An*. *funestus* and *An*. *gambiae* from northern Cameroon (Mandeng et al., 2019; Menze et al., 2018, 2020). In a study conducted at Gounougou in 2003, high resistance to permethrin was observed in *An. gambiae* s.l. with mortality of 34.7% (Ndjemaï et al., 2009). Over a decade later, we reported a further significant decrease in mortality to permethrin at 3.75% in Gounougou *An. coluzzii* populations in 2017 (Fadel et al., 2019) and by 2019 no mortality at all. The high resistance (33% mortality) even at 10× permethrin concentration, is higher than that measured in the same species from Massakory (10× permethrin mortality of 55.5%) in the central Sahel region of Chad (Ibrahim, Fadel, Tchouakui, et al., 2019); in *An. gambiae* s.l. from Lagos and Ogun (10× permethrin mortalities at 89.0% and 95.0%, respectively) in Nigeria (Awolola et al., 2018); and in *An. gambiae* s.l. from Guinea‐Bissau (10× permethrin mortality at 86.0%) (Silva et al., 2020). These findings suggest that the high pyrethroid resistance intensity could negatively impact the efficacy of pyrethroid‐impregnated bed nets, the primary tool for malaria control in Gounougou.

The finding of marginal resistance to bendiocarb contrasts with previous observations for the Gounougou population (Antonio‐Nkondjio et al., 2008; Fadel et al., 2019) and suggests emerging carbamate resistance in northern Cameroon, which should be studied further. This moderate bendiocarb resistance has been reported in *An. gambiae* s.l. from other regions of Cameroon (Antonio‐Nkondjio et al., 2016). Also, studies carried out in Sahel from Nigeria and Niger in West Africa found *An. coluzzii* exhibited comparable moderate bendiocarb resistance (Ibrahim, Mukhtar, Datti, et al., 2019; Ibrahim, Mukhtar, Irving, et al., 2019). Similar patterns of marginal resistance to bendiocarb have been recently observed in *An*. *coluzzii* from N'Djamena in Central Sahel of Chad (Ibrahim, Fadel, Tchouakui, et al., 2019). The development of bendiocarb resistance could be due to additional selection pressure imposed by carbamate‐based pesticide use in agriculture for crop protection in northern Cameroon (Menze et al., 2018), as carbamate‐based IRS has not been implemented here (Antonio‐Nkondjio et al., 2017). In support of this, mosquitoes originating from cultivated breeding sites were more resistant to bendiocarb than those sampled from uncultivated sites in Central Cameroon (Antonio‐Nkondjio et al., 2016).

The combination of bendiocarb resistance and full susceptibility to malathion suggest no cross resistance between these insecticide classes and point to a metabolic, rather than target‐site, resistance mechanism, potentially driven by the overexpression of cytochrome P450s such as *CYP6M2* (Edi et al., 2014), in the absence of the G119 *ace*‐1 mutation in the Gounougou population. Full susceptibility to malathion is in line with previous studies (Antonio‐Nkondjio et al., 2008; Fadel et al., 2019; Menze et al., 2018; Ndjemaï et al., 2009) and indicates that organophosphates remain suitable alternative insecticides for IRS in this region.

### 4.3 Synergist assays and transcriptional profiling suggest metabolic mechanisms contributing to highly intense resistance

Pre‐exposure to DEM significantly restored partial susceptibility to both permethrin and DDT, which is in line with GST‐mediated metabolic resistance (Enayati et al., 2005; Riveron et al., 2014). Synergist bioassays with DEM have been shown in several studies to partially restore insecticide susceptibility in *An. funestus* and *Aedes* mosquitoes (Tchouakui et al., 2019; Yougang et al., 2020) from Cameroon. Significant recovery of susceptibility to 5× permethrin and 10× permethrin on pre‐exposure to PBO are indicative of cytochrome P450‐driven permethrin resistance escalation. However, the failure to recover susceptibility in the PBO assay with 1× permethrin suggests considerable loss of efficacy of this synergist and/or alternative mechanisms conferring complementary resistance, such as *GSTs*, at low doses of permethrin. Indeed this finding is in line with the previous result of a considerable loss of efficacy of the PBO–permethrin containing Olyset Plus bed nets seen in *An*. *coluzzii* from Gounougou in 2017 (Fadel et al., 2019), in *An*. *coluzzii* from Chad (Ibrahim, Fadel, Tchouakui, et al., 2019), in both *An. gambiae* and *An. funestus* from Mibellon in Cameroon (Menze et al., 2018), and in *An. funestus* from Democratic Republic of Congo (Riveron, Watsenga, et al., 2018) and Mozambique (Riveron et al., 2019).

Differences in gene expression patterns were assessed by comparing permethrin‐exposed, resistant field mosquitoes, control mosquitoes (from the same population but unexposed), and females from the susceptible Ngousso colony. The comparative RNA‐seq‐based transcription profiling between resistance phenotype backgrounds highlighted significant differences in the level of expression of several resistance genes, including *GSTe2* (*AGAP009194*) and *CYP6Z2* (*AGAP008218*) which are highly expressed when comparing both permethrin‐alive and unexposed versus susceptible mosquitoes, respectively. This is supported by the PCA analysis that revealed a strong positive correlation between potential resistance genes expression pattern and resistance phenotype. This difference in gene expression is in line with previous reports in *An*. *gambiae* s.l. (Bonizzoni et al., 2015; Simma et al., 2019) and in *An*. *funestus* across Africa (Mugenzi et al., 2019; Riveron et al., 2017; Weedall et al., 2019). Indeed, the above two genes have been shown to be the major drivers of pyrethroid resistance across the Sahel regions of four sub‐Saharan African countries (Nigeria, Niger, Chad, and Cameroon; Ibrahim et al., 2023). The finding that the *SAP2* gene was overexpressed in Gounougou *Anopheles* is in agreement with a previous report that the gene was significantly upregulated, which is in agreement with its positive directional selection in *An*. *gambiae* from Cameroon (Ingham et al., 2020), suggesting that *SAP2* plays a role in conferring permethrin resistance in the Gounougou *An. coluzzii* population. qRT‐PCR of the Gounougou population revealed upregulation of seven major metabolic resistance genes compared with the fully susceptible Ngousso colony. The most consistently overexpressed gene in mosquitoes exposed to different concentrations of permethrin was *GSTe2* (AGAP009194), which has been shown to be not only a major DDT metabolizer in *An*. *gambiae* population (Ibrahim et al., 2023; Mitchell et al., 2014) but is also able to confer cross resistance to permethrin in *An*. *funestus* (Riveron et al., 2014) and *An. coluzzii* (Ibrahim et al., 2023). In addition, six P450s were also found to be upregulated. These include *CYP6Z2* (AGAP008218) shown to be involved in resistance to primary products of hydrolysis of pyrethroids (McLaughlin et al., 2008) and resistance to pyriproxyfen (Yunta et al., 2016), *CYP6Z1* (AGAP008219) previously shown to be capable of metabolizing DDT (Chiu et al., 2008) and recently found to be overexpressed in resistant *An*. *coluzzii* in individuals from Mali following sublethal permethrin exposure (Main et al., 2018); the well‐known pyrethroid metabolizer *CYP6P3* (AGAP002865; Edi et al., 2014; Müller et al., 2008), *CYP6M2* (AGAP008212) known to metabolize deltamethrin (Stevenson et al., 2011), *CYP4G16* (AGAP001076) known to catalyze epicuticular hydrocarbon biosynthesis in *An*. *gambiae* and is involved in cuticular resistance (Balabanidou et al., 2016), and *CYP4G17* (AGAP000877) gene also implicated in cuticular resistance (Kefi et al., 2019).

However, qRT‐PCR with *SAP 2* failed to confirm the upregulation of this gene, contrary to RNA‐seq results. This discrepancy in expression between RNA‐seq and qRT‐PCR could be explained by the fact this gene in *An*. *coluzzii* from Gounougou could be subjected to alternative splicing, as recently suggested (Zhang et al., 2019), or that polymorphisms in primer binding sites could have impacted the qRT‐PCR assays. Further studies to resolve this inconsistency in gene expression quantification are needed. However, the low overexpression of *SAP1* and *SAP3* found in this study is similar to a trend previously observed in a pyrethroid resistant *An*. *coluzzii* population from Tiassalé, Burkina Faso (Ingham et al., 2020).

### 4.4 Detection of signatures of selective sweeps

In this study, the findings of high frequencies of 2La, 2Rb, and 2Rc inversion polymorphisms in the field *An*. *coluzzii*, compared with Ngousso colony, suggested strong phenotypic adaptation in this species in the farmed Guinea Savannah region of Cameroon. Most notably, in addition to several of the major classes of insect cuticular protein genes (chitinases, chitin synthase, *CPR*, *CPAP* proteins) that have been described to play potentially a crucial role in insecticide resistance in *An*. *coluzzii* (Toe et al., 2015), several other genes previously implicated in detoxification and metabolic insecticide resistance in *Anopheles* and other mosquitoes, and which were highly upregulated genes in this study, sit within these inversions. For example, the chymotrypsin genes *CHYM1* and *CHYM2* located in the 2La region, known to defend insects against plants' proteinase inhibitor (Dunse et al., 2010) and previously shown to be overexpressed in insecticide resistant populations of *An*. *gambiae* and *An*. *coluzzii* (Antonio‐Nkondjio et al., 2016; Toe et al., 2015), were among the top 50 most overexpressed genes. The *CPAP3‐A1b* gene (AGAP000987) has been shown to be highly overexpressed in deltamethrin‐resistant populations of *An*. *coluzzii* from Burkina Faso (Toe et al., 2015). The chitinases *Cht24* and *Cht6*, previously shown to be overexpressed in a resistant population of Ethiopian *An*. *arabiensis* (Messenger et al., 2021), are found to be overexpressed in this study and sit in the 2La inversion. Carbonic anhydrase I (AGAP013402) and a lipase (AGAP002353) linked to deltamethrin resistance in *Culex pipiens pallen* (Hu et al., 2020) are both overexpressed in the Gounougou population of *An*. *coluzzii* and both sit within the 2Rb inversion.

### 4.5 Multiple *kdr* mutations are present in the Gounougou *An. coluzzii* population

TaqMan genotyping detected the presence of the 1014F *kdr* mutation at a higher frequency, whereas the 1014S and 1575Y mutations were detected in lower frequencies. The moderate frequency of the 1014F mutation is not consistent with that of a previous report from this study site (Fadel et al., 2019), where the mutation was found at a higher frequency (65.25%). This decrease in L1014F frequency from 2017 to 2019 could be explained by the fact despite the global trends, the local practices in Gounougou, both in farming and public health vector control tool uses, could speedily impact the dynamics of the pyrethroid/DDT resistance allele. In addition, this finding is suggesting that 1014F resistant allele is subjected to its fitness cost if even an increased role of other major non‐P450 detoxification genes driving the phenotypic pyrethroid/DDT resistance in the field are present. Significant decrease in *kdr* mutation was also reported in some populations of *An. gambiae* s.l. in Benin. (Assogba et al., 2020). Contrary to the observation in 2019, in 2017 we observed higher frequencies of the 1014S *kdr* mutation at Gounougou. Similar trends have been reported in Nigeria. For example, in 2014 a single *An*. *arabiensis* with heterozygote L1014S mutation was identified out of 26 (Ibrahim et al., 2014), and 5 years late no L1014S mutation was observed in the same locality (Ibrahim, Mukhtar, Datti, et al., 2019). Lower frequencies of the 1014S and 1575Y mutations in *An. coluzzii* have been also described recently in Cameroon (Bamou et al., 2019; Mandeng et al., 2019), in Chad (Ibrahim, Fadel, Tchouakui, et al., 2019), in Togo (Djegbe et al., 2018), and in Côte d'Ivoire (Edi et al., 2017; Mouhamadou et al., 2019) suggesting more recent occurrence of these mutations in *An*. *coluzzii* in West and Central Africa. There was no significant decrease in the frequency of the 1014S mutation in F_0_ females from the 2019 collection compared with the 2017 collection, with no homozygote‐resistant sample detected for the 2 years investigated, consistent with a fitness cost linked with the homozygosity of this mutation as hypothesized previously (Ibrahim, Mukhtar, Datti, et al., 2019).

On the contrary, there was a significant decrease in the 1575Y mutation frequency in *An*. *coluzzii* samples collected in 2019 than those sampled in 2017. Only 7% of double mutant females (homozygote resistant) harboring both 1014F and 1575Y were found in both collections from 2017 and 2019. The fact that all homozygote‐resistant individuals for 1575Y are found also to be homozygote resistant for 1014F established association between the 1014F and 1575Y mutations, suggesting the presence of the 1014F‐1575Y haplotype which is thought to boost pyrethroid and DDT resistance, by compensating the deleterious fitness effect of the 1014F homozygote as previously shown in West Africa (Jones et al., 2012).

The distribution of frequency of 1014F mutation was only assessed in individuals surviving at different permethrin concentrations. Unfortunately, it was not possible to establish a correlation between the *kdr* genotypes and pyrethroid/DDT resistance phenotypes due to high mortalities with permethrin and DDT. Nevertheless, it seems that there was a reduction of 1014F frequency from 1× to 10× suggesting that *kdr* plays only a minor role in the escalation of resistance to permethrin. This is also supported by PBO and DEM synergist results which show a greater recovery of susceptibility when mosquitoes were exposed to 5× and 10× but less with 1×. This observation suggests that the molecular drivers of resistance escalation in these mosquitoes are different to those driving resistance to standard discriminating concentration of insecticides.

## 5 CONCLUSION

This study revealed a marked escalation of permethrin resistance and budding bendiocarb resistance in a population of the major malaria vector *An. coluzzii* from northern Cameroon. This poses a threat to malaria control using bed nets and to future implementation of control interventions. Several well‐characterized cytochrome P450s and *GSTe2* were overexpressed in the resistant mosquitoes, suggesting that metabolic resistance is the predominant resistance mechanism in this population and potentially across Sudan Savannah of northern Cameroon. These findings should be taken into consideration in planning future malaria control interventions in this region.

## CONFLICT OF INTEREST STATEMENT

The authors declare no financial and ethical conflicts of interest.

## Supporting information


Figure S1.

File S1.

File S2.

File S3.

## Data Availability

The dataset(s) supporting the conclusions of this article are available in the European Nucleotide archive, Project PRJEB51644 (https://www.ebi.ac.uk/ena/browser/view/PRJEB51644).
