# Supplementary figures and images for: Exploring the molecular mechanisms of increased intensity of pyrethroid resistance in Central African population of a major malaria vector Anopheles coluzzii
Source: Evol Appl. 2024 Feb 26;17(2):e13641. doi: 10.1111/eva.13641 (PMC10895554; doi:10.1111/eva.13641)

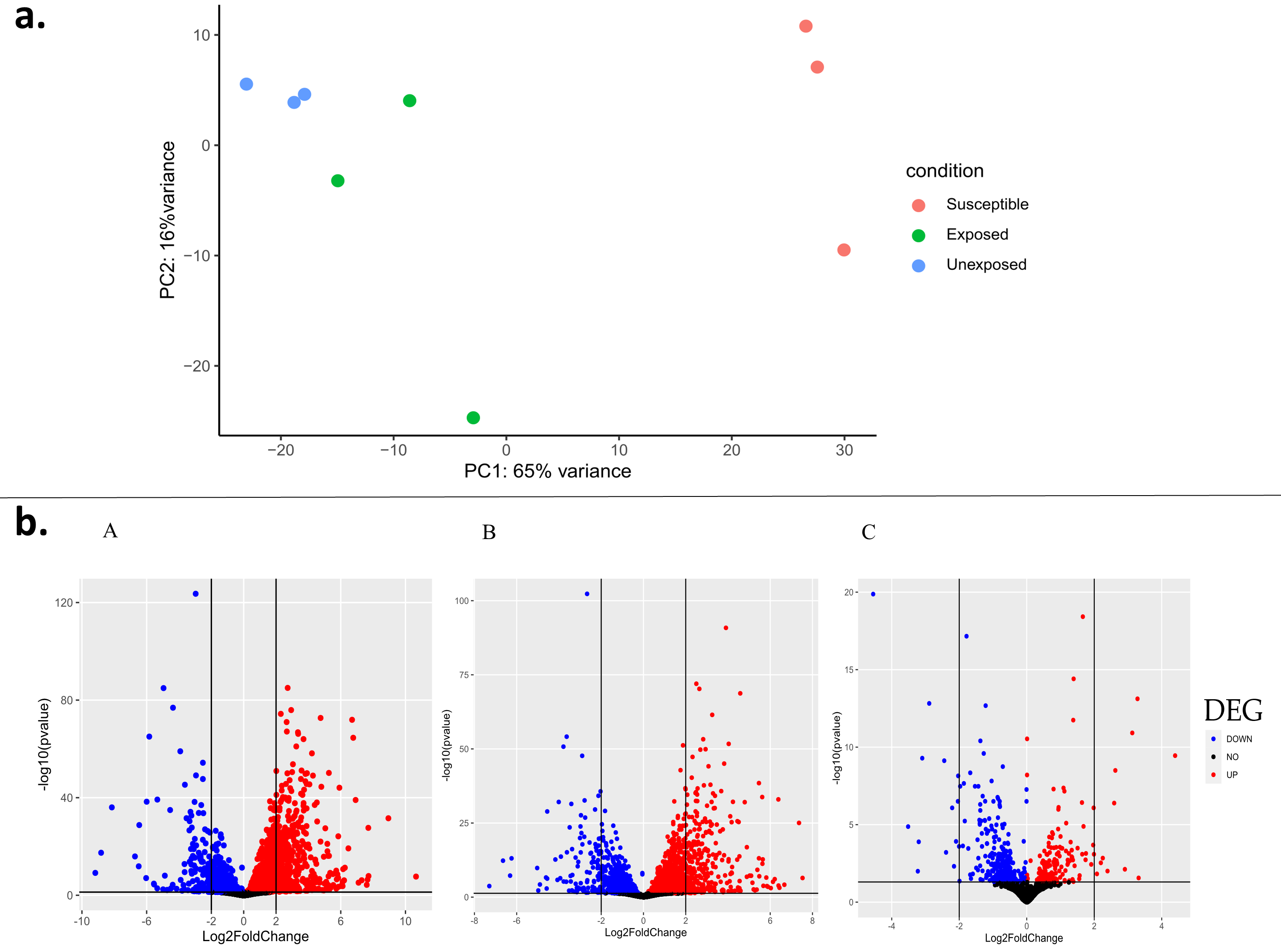

Supplement: Supplementary file 1 — Figure S1. [file EVA-17-e13641-s004.tif]
