# Supplementary material for: Exploring the molecular mechanisms of increased intensity of pyrethroid resistance in Central African population of a major malaria vector Anopheles coluzzii
Source: Evol Appl. 2024 Feb 26;17(2):e13641. doi: 10.1111/eva.13641 (PMC10895554; doi:10.1111/eva.13641)
Supplement: Supplementary file 2 — File S1. [file EVA-17-e13641-s002.docx]

**Article title**:

**Exploring the molecular mechanisms of increased intensity of pyrethroid resistance in Central African population of a major malaria vector *Anopheles coluzzii***

**R-code**, DESeq2 Rscript file

**## Command used to run this script "Rscript ./GOUN.DESeq2.Sept2019.R Anopheles-gambiae-PEST_TRANSCRIPTS_AgamP4.10.fa.tx2gene Angambiae GOUN NGOUSSO"**

**#**!/usr/bin/env Rscript

library(tximport)

library(readr)

library(DESeq2)

library(ggplot2)

library(IHW)

library(RColorBrewer)

library(gplots)

library(hexbin)

library(pheatmap)

library(apeglm)

library(ggplot2)

args <- commandArgs(trailingOnly = TRUE)

GeneMapFile = args[1]

Species= args[2]

Experiment = args[3]

Control = args[4]

TestDir=paste("./*-",Experiment,"*",Species,".quants", sep="")

CtrlDir=paste("./*",Control,"*",Species,".quants", sep="")

ExpDir=c(TestDir,CtrlDir)

samples <- Sys.glob(ExpDir)

prefix=Experiment

files <- file.path(samples, "quant.sf")

names <- gsub("./", "",samples)

names <- gsub(".quants", "",names)

sampsName<- paste(Experiment,".samples.txt",sep="")

samps <- read.table(sampsName,header=T,sep="\t", row.names=1)

samps$condition<- factor(samps$condition)

table(samps$condition)

names(files) <- rownames(samps)

names(files) <- sub ("^X", "", names(files))

rownames(samps) <- sub ("^X", "", rownames(samps))

tx2gene<-read.table(GeneMapFile,header=T)

txi <- tximport(files, type = "salmon", tx2gene = tx2gene)

names(txi)

head(txi$counts)

colnames(txi$counts) <- sub ("^X", "", colnames(txi$counts))

Counts<- paste(prefix,".salmon.matrix.csv",sep="")

write.csv(txi$counts,file=Counts)

all(rownames(samps) %in% colnames(txi$counts))

all(rownames(samps) == colnames(txi$counts))

**##Level is Susceptible for comparisons**

dds <- DESeqDataSetFromTximport(txi, colData = samps, design = ~ condition)

keep <- rowSums(counts(dds)) >= 10

dds <- dds[keep,]

dds$condition <- relevel(dds$condition, ref = "Susceptible")

dds<-DESeq(dds)

NCounts<- paste(prefix,".salmon.matrix.normalised.csv",sep="")

write.csv(round((counts(dds, normalized=TRUE)),0),file=NCounts)

resultsNames(dds)

resEvsS <- lfcShrink(dds, coef="condition_Exposed_vs_Susceptible", type="apeglm", lfcThreshold=1)

resEvsSOrdered<-resEvsS[order(resEvsS$svalue),]

resUvsS <- lfcShrink(dds, coef="condition_Unexposed_vs_Susceptible", type="apeglm", lfcThreshold=1)

resUvsSOrdered<-resUvsS[order(resUvsS$svalue),]

resEvsSSig <- subset(resEvsSOrdered, svalue < 0.005)

resUvsSSig <- subset(resUvsSOrdered, svalue < 0.005)

write.csv(as.data.frame(resEvsSSig),file=paste(prefix,"EvsS.apeglm.Sig005.lfc1.csv",sep="."))

write.csv(as.data.frame(resUvsSSig),file=paste(prefix,"UvsS.apeglm.Sig005.lfc1.csv",sep="."))

write.csv(as.data.frame(resEvsSOrdered),file=paste(prefix,"EvsS.apeglm.lfc1.csv",sep="."))

write.csv(as.data.frame(resUvsSOrdered),file=paste(prefix,"UvsS.apeglm.lfc1.csv",sep="."))

resEvsS_lfc0 <- lfcShrink(dds, coef="condition_Exposed_vs_Susceptible", type="apeglm")

resEvsS_lfc0.Ordered<-resEvsS_lfc0[order(resEvsS_lfc0$padj),]

resEvsSSig_lfc0 <- subset(resEvsS_lfc0.Ordered, padj < 0.05)

write.csv(as.data.frame(resEvsSSig_lfc0),file=paste(prefix,"EvsS.apeglm.P-val0.05.lfc0.csv",sep="."))

write.csv(as.data.frame(resEvsS_lfc0.Ordered),file=paste(prefix,"EvsS.apeglm.lfc0.csv",sep="."))

resUvsS_lfc0 <- lfcShrink(dds, coef="condition_Unexposed_vs_Susceptible", type="apeglm")

resUvsS_lfc0.Ordered<-resUvsS_lfc0[order(resUvsS_lfc0$padj),]

resUvsSSig_lfc0 <- subset(resUvsS_lfc0.Ordered, padj < 0.05)

write.csv(as.data.frame(resUvsSSig_lfc0),file=paste(prefix,"UvsS.apeglm.P-val0.05.lfc0.csv",sep="."))

write.csv(as.data.frame(resUvsS_lfc0.Ordered),file=paste(prefix,"UvsS.apeglm.lfc0.csv",sep="."))

pdf(file=paste(prefix,"EvsS.MA.pdf",sep="."))

plotMA(resEvsS_lfc0, ylim=c(-3,3), cex=.8)

plotMA(resEvsS, ylim=c(-3,3), cex=.8)

abline(h=c(-1,1), col="dodgerblue", lwd=2)

dev.off()

pdf(file=paste(prefix,"UvsS.MA.pdf",sep="."))

plotMA(resUvsS_lfc0, ylim=c(-3,3), cex=.8)

plotMA(resUvsS, ylim=c(-3,3), cex=.8)

abline(h=c(-1,1), col="dodgerblue", lwd=2)

dev.off()

**##Re-level to test Exposed vs Unexposed**

ddm <- DESeqDataSetFromTximport(txi, colData = samps, design = ~ condition)

keep <- rowSums(counts(ddm)) >= 10

ddm <- ddm[keep,]

ddm$condition <- relevel(ddm$condition, ref = "Unexposed")

ddm<-DESeq(ddm)

resultsNames(ddm)

resEvsU <- lfcShrink(ddm, coef="condition_Exposed_vs_Unexposed", type="apeglm", lfcThreshold=1)

resEvsUOrdered<-resEvsU[order(resEvsU$svalue),]

resEvsUSig <- subset(resEvsUOrdered, svalue < 0.005)

write.csv(as.data.frame(resEvsUSig),file=paste(prefix,"EvsU.apeglm.Sig005.lfc1.csv",sep="."))

write.csv(as.data.frame(resEvsUOrdered),file=paste(prefix,"EvsU.apeglm.lfc1.csv",sep="."))

resEvsU_lfc0 <- lfcShrink(ddm, coef="condition_Exposed_vs_Unexposed", type="apeglm")

resEvsU_lfc0.Ordered<-resEvsU_lfc0[order(resEvsU_lfc0$padj),]

resEvsUSig_lfc0 <- subset(resEvsU_lfc0.Ordered, padj < 0.05)

write.csv(as.data.frame(resEvsUSig_lfc0),file=paste(prefix,"EvsU.apeglm.P-val0.05.lfc0.csv",sep="."))

write.csv(as.data.frame(resEvsU_lfc0.Ordered),file=paste(prefix,"EvsU.apeglm.lfc0.csv",sep="."))

pdf(file=paste(prefix,"EvsU.MA.pdf",sep="."))

plotMA(resEvsU_lfc0, ylim=c(-3,3), cex=.8)

plotMA(resEvsU, ylim=c(-3,3), cex=.8)

abline(h=c(-1,1), col="dodgerblue", lwd=2)

dev.off()

**Table S1.** Primers used for qRT-PCR

| **Gene Name** | **Gene ID** | **Oligo Name** | **Primer sequence (5’-3’)** | **qRT-PCR efficiency** |
| --- | --- | --- | --- | --- |
| *GSTe2* | AGAP009194 | qpColz_GSTe2F  qpColz_GSTe2R | ACCATTAATCTGCTAACGGGTG  AATTTACACGGGCCTGCTTG | 1.033 |
| *CYP6Z1* | AGAP008219 | CYP6Z1F_F  CYP6Z1_R | CCCGCAACTGTATCGGTCTG  TTCGGTGCCAGTGTGATTGA | 0.986 |
| *CYP6Z2* | AGAP008218 | qpColz_CYP6Z2F  qpColz_CYP6Z2R | AGGCCACGAAGAACTACGAT  ACTTTTGCAGGAGTTGTGGC | 1.061 |
| *CYP6P3* | AGAP002865 | qpColz_CYP6P3F  qpColz_CYP6P3R | AGCGGCTGAGAGAGGAAATT  GCTTCGGGATCACATGCTTT | 0.973 |
| *CYP6M2* | AGAP008212 | qpColz_CYP6M2F  qpColz_CYP6M2R | AGGTCGTGAGTGTGTGAGAG  CTTTCGAAGCCACACGGAAA | 0.99 |
| *CYP4G16* | AGAP001076 | qpColz_CYP4G16F  qpColz_CYP4G16R | ACTCCCTTTGATTGGAAACGC  TCAGATGGAAAGTAGGGGCA | 1,008 |
| *CYP4G17* | AGAP000877 | qpColz_CYP4G17F  qpColz_CYP4G17R | TGTCACGACTACATGAGCGA  CGCAGGTGGATCTTCAGTTG | 0.908 |
| *SAP1* | AGAP008052 | qpColz_SAP1F  qpColz_SAP1R | TTTGATCCGGAGAACAAGTAC  CGTTCTTCCGGGTTTCCA | 107.6 |
| *SAP2* | AGAP008051 | qpColz_SAP2F  qpColz_SAP2R | CTACTTCAAGTGCCTGATGG  CTTGATGCCCTCCTTCTTG | 103.1 |
| *SAP3* | AGAP008054 | qpColz_SAP3F  qpColz_SAP3R | GAGAAGCAGAAGAGTGGCAC  ACAGGTTGATGCCCTTCTTC | 99.6 |
| *Elongation factor* | AGAP005128 | EF_F  EF_R | GGCAAGAGGCATAACGATCAATGCG  GTCCATCTGCGACGCTCCGG | 0.979 |
| *RPS7* | AGAP010592 | RPS7_F  RPS7_R | CCACCATCGAACACAAAGTTGA  TGCTGCAAACTTCGGCTATTC | 0.897 |

**Table S2.** RNA-seq sample summary of the alignment statistics.

| **Sample ID** | **Reads to align**  **(R1+R2)** | **Aligned reads (%) ^1^** | | **Aligned reads, filtered (%) ^1,2^** | **Aligned in pair (%) ^3^** | **Singleton (%) ^3^** |
| --- | --- | --- | --- | --- | --- | --- |
| Susceptible_R1 | 22935376 | 21380943 (93.22) | 21380943 (93.22) | | 21061238 (98.50) | 319705 (1.50) |
| Susceptible_R2 | 21521594 | 20186461 (93.8) | 20186461 (93.8) | | 19899040 (98.58) | 287421 (1.42) |
| Susceptible_R3 | 35607160 | 32568174 (91.47) | 32568174 (91.47) | | 32054416 (98.42) | 513758 (1.58) |
| Resistant_R1 | 24924672 | 23402379 (93.89) | 23402379 (93.89) | | 23002742 (98.29) | 399637 (1.71) |
| Resistant_R2 | 24507610 | 23055920 (94.08) | 23055920 (94.08) | | 22683092 (98.38) | 372828 (1.62) |
| Resistant_R3 | 29030956 | 27275670 (93.95) | 27275666 (93.95) | | 26831596 (98.37) | 444070 (1.63) |
| Control_R1 | 25156554 | 23665448 (94.07) | 23665447 (94.07) | | 23299917 (98.46) | 365530 (1.54) |
| Control_R2 | 24938738 | 23091144 (92.59) | 23091144 (92.59) | | 22720240 (98.39) | 370904 (1.61) |
| Control_R3 | 21633948 | 20039292 (92.63) | 20039292 (92.63) | | 19723450 (98.42) | 315842 (1.58) |

^1^ % of reads to align,^2^ Aligned reads filtered to remove reads with mapping quality <10; ^3^ % of filtered aligned reads with both read and its mate mapped to opposing strands of the reference sequence, with 3' ends innermost and 5' ends within the allowed distance from each other (0-500 bp). R, replicate, Susceptible, Ngousso, Resistant, Gounougou *An. coluzzii* permethrin-alive, Control, Gounougou *An. coluzzii* unexposed.

**Table S3:** common top 50 genes differentially and constitutively upregulated in Gounougou *An*. *coluzzii* at FDR-adjusted and p <0.05.

| Gene ID | R-S | C-S | Gene Description |
| --- | --- | --- | --- |
| AGAP012833 | 6.21 | 6.21 | 28S ribosomal protein mitochondrial, mRpS24 |
| AGAP004802 | 3.01 | 3.82 | 4-hydroxyphenylpyruvate dioxygenase |
| AGAP001124 | 4.44 | 4.95 | Aminomethyltransferase |
| AGAP007035 | 3.82 | 4.75 | Anopheles Plasmodium-responsive Leucine-Rich Repeat 1B, APL1B |
| AGAP013402 | 5.03 | 6.15 | carbonic anhydrase I |
| AGAP005372 | 3.47 | 3.15 | carboxylesterase beta esterase, COEBE3C |
| AGAP013509 | 2.72 | 3.74 | carboxylesterase clade H 2C member 1 |
| AGAP011228 | 4.50 | 5.91 | cathepsin B precursor |
| AGAP006191 | 5.62 | 5.04 | chitinase, Cht24 |
| AGAP006709 | 4.17 | 6.47 | chymotrypsin, CHYM1 |
| AGAP006710 | 2.69 | 4.80 | chymotrypsin, CHYM2 |
| AGAP006711 | 4.11 | 6.70 | chymotrypsin, CHYM3 |
| AGAP009217 | 6.39 | 5.84 | CLIP-domain serine protease, CLIPB12 |
| AGAP009215 | 1.45 | 4.64 | CLIP-domain serine protease, CLIPB18 |
| AGAP008091 | 3.82 | 3.73 | CLIP-domain serine protease, CLIPE1 |
| AGAP012731 | 3.79 | 4.22 | COMPASS component SWD3 |
| AGAP007408 | 3.75 | 5.23 | C-type lectin - mannose binding, CTLMA8 |
| AGAP005526 | 7.36 | 7.70 | Cubilin |
| AGAP003375 | 3.40 | 3.93 | cuticular protein, CPR114 |
| AGAP010123 | 6.39 | 6.92 | cuticular protein, CPR131 |
| AGAP009868 | 4.47 | 6.11 | cuticular protein, CPR73 |
| AGAP006425 | 2.66 | 4.68 | cyanogenic beta-glucosidase |
| AGAP006422 | 3.29 | 3.91 | cyanogenic beta-glucosidase |
| AGAP001116 | 4.03 | 4.75 | D-amino-acid oxidase |
| AGAP005500 | 4.18 | 4.61 | dehydrogenase/reductase SDR family member 11 precursor |
| AGAP003600 | 3.25 | 3.37 | Elongation of very long chain fatty acids protein |
| AGAP028931 | 6.13 | 7.72 | Eukaryotic large subunit ribosomal RNA, LSU_rRNA_eukarya |
| AGAP028918 | 3.76 | 5.25 | Eukaryotic large subunit ribosomal RNA, LSU_rRNA_eukarya |
| AGAP012000 | 2.86 | 3.93 | fibrinogen and fibronectin |
| AGAP006914 | 2.36 | 3.93 | fibrinogen-related protein 1 |
| AGAP010531 | 3.59 | 4.07 | fibrinogen-related protein 7 |
| AGAP010399 | 4.12 | 2.98 | Flavin-containing monooxygenase FMO GS-OX-like 1 |
| AGAP012201 | 4.55 | 5.80 | Histone H2B |
| AGAP012550 | 5.89 | 6.12 | kelch-like protein 28 |
| AGAP005496 | 3.76 | 3.48 | leucine-rich immune protein (Short), LRIM12 |
| AGAP028028 | 3.68 | 4.48 | leucine-rich immune protein (TM), LRIM16A |
| AGAP028064 | 3.33 | 4.06 | leucine-rich immune protein (TM), LRIM16B |
| AGAP002353 | 2.94 | 3.39 | lipase |
| AGAP000184 | 3.08 | 3.70 | malate dehydrogenase |
| AGAP010057 | 4.54 | 5.18 | nicotinic acetylcholine receptor beta-2 subunit |
| AGAP004978 | 3.21 | 3.90 | prophenoloxidase 9, PPO9 |
| AGAP013155 | 5.39 | 5.34 | Protease m1 zinc metalloprotease |
| AGAP004954 | 3.28 | 3.75 | rhythmically expressed gene 2 protein |
| AGAP008052 | 5.47 | 3.88 | sensory appendage protein, SAP2 |
| AGAP012696 | 5.68 | 7.66 | Sulfotransferase (Sult) |
| AGAP010831 | 6.03 | 3.93 | thioester-containing protein, TEP8 |
| AGAP001002 | 4.08 | 5.72 | Toll protein |
| AGAP012083 | 3.38 | 4.30 | Trafficking protein particle complex 5 |
| AGAP010367 | 3.63 | 3.85 | UDP-N-acetyl-alpha-D-galactosamine |
| AGAP012818 | 3.96 | 4.23 | V-type proton ATPase subunit a |

**Table S4:** list of most common detoxification and metabolic genes differentially and constitutively downregulated in Gounougou An. *coluzzii* at FDR-adjusted and p <0.05.

| Gene ID | R-S | C-S | Gene Description |
| --- | --- | --- | --- |
|  |  | **Carboxylesterases** |  |
| AGAP003115 | -0.38 | -1.12 | carboxylesterase, COE2580 |
| AGAP006728 | -0.44 | -2.18 | carboxylesterase, COEAE7G |
| AGAP011916 | -0.42 | -1.07 | carboxylesterase, COEB6582 |
|  |  | **Cytochrome P450** |  |
| AGAP009375 | -0.19 | -1.89 | cytochrome P450, CYP9M2 |
| AGAP008212 | -0.76 | -1.80 | cytochrome P450, CYP6M2 |
| AGAP005658 | -1.32 | -2.13 | cytochrome P450, CYP15B1 |
| AGAP013490 | -3.41 | -3.34 | cytochrome P450, CYP4H24 |
| AGAP003343 | -0.83 | -1.32 | cytochrome P450, CYP6AG1 |
| AGAP010961 | -2.13 | -2.52 | Cytochrome P450, CYP6AK1 |
| AGAP008214 | -1.51 | -2.09 | cytochrome P450, CYP6M4 |
| AGAP002866 | -2.83 | -5.55 | cytochrome P450, CYP6P5 |
|  |  | **Glutathion S-Transferases** |  |
| AGAP004381 | -0.53 | -3.14 | glutathione S-transferase, GSTd4 |
| AGAP003257 | -1.86 | -1.22 | glutathione S-transferase, GSTU2 |
| AGAP010814 | -6.24 | -3.66 | thioester-containing protein, TEP6 |
|  |  | **Transporters** |  |
| AGAP006379 | -0.76 | -1.36 | transporter, ABCA1 |
| AGAP012155 | -1.13 | -0.52 | transporter, ABCA6 |
| AGAP012156 | -2.39 | -1.76 | transporter. ABCAS |
| AGAP027980 | -0.75 | -1.19 | transporter,ABCC10 |
| AGAP008436 | -1.35 | -0.59 | transporter,ABCC11 |
| AGAP009799 | -1.46 | -2.09 | transporter, ABCC13 |
| AGAP008437 | -1.19 | -1.84 | transporter, ABCC8 |
| AGAP028128 | -1.55 | -2.05 | transporter, ABCC9 |
|  |  | **Other detox genes** |  |
| AGAP008294 | -2.94 | -3.02 | trypsin, TRYP3 |
| AGAP008292 | -2.39 | -4.55 | Trypsin,TRYP4 |
| AGAP008291 | -4.17 | -6.46 | trypsin,TRYP5 |
| AGAP008290 | -4.56 | -8.82 | trypsin, TRYP6 |
| AGAP008293 | -3.45 | -8.15 | trypsin, TRYP7 |
| AGAP009022 | -0.05 | -1.01 | chitinase, Cht9 |
| AGAP006148 | 0.28 | -1.49 | cuticular protein,CPLCA3 |
| AGAP008465 | -3.15 | -4.98 | cuticular protein, CPLG22 |
| AGAP010122 | -1.30 | -0.39 | cuticular protein, CPR132 |
| AGAP005995 | -0.84 | -1.03 | cuticular protein, CPR138 |
| AGAP006369 | -0.35 | -1.78 | cuticular protein, CPR144 |
| AGAP006000 | -1.03 | -0.46 | cuticular protein, CPR25 |
| AGAP001669 | -3.62 | -3.24 | cuticular protein, CPR6 |

**Table S5:** Ploidy scores and frequencies of chromosomal inversion polymorphisms

| **Ploidy Score (Frequency)** | | | | | | |  |
| --- | --- | --- | --- | --- | --- | --- | --- |
| Population Inversions | | | | | | |  |
|  | 2La | 2Rb | 2Rc | 2Rd | 2Rj | 2Ru | |
| Gounougou | 15.877 (0.992) | 10.039 (0.627) | 10.083 (0.630) | 0.791 (0.0494) | 2.583 (0.1614) | 1.583 (0.0989) | |
| Ngousso | 1.114 (0.069) | 0.714 (0.0446) | 1.583 (0.0989) | 0.166 (0.0104) | 8.5 (0.53125) | 0.00 | |

Ploidy score = average score for all replicates from the same population; frequency is the ratio of the ploidy score in relation to the total of the possible ploidy (16).

**Table S6:** Temporal variation in allele and genotype frequencies at the *kdr* mutation locus in *An*. *coluzzii* populations from Gounougou.

|  |  | **Alleles** | | |  | **Genotypes** | | |
| --- | --- | --- | --- | --- | --- | --- | --- | --- |
| Year | *kdr* locus | N | f(R) | f(S) |  | RR (%) | RS (%) | SS (%) |
| 2017 | L1014F* | 59 | 0.6525 | 0.3475 |  | 25 (42.37) | 27 (45.76) | 7 (11.87) |
|  | L1014S | 42 | 0.1547 | 0.8453 |  | 0 (0) | 13 (30.95) | 29 (69.05) |
|  | N1575Y | 71 | 0.5140 | 0.4860 |  | 9 (12.68) | 55 (77.46) | 7 (9.86) |
| 2019 | L1014F | 68 | 0.50 | 0.50 |  | 12(17.64) | 44 (64.72) | 12 (17.64) |
|  | L1014S | 68 | 0.0661 | 0.9339 |  | 0 (0) | 9 (13.23) | 59 (86.77) |
|  | N1575Y | 68 | 0.1029 | 0.8961 |  | 1 (1.48) | 12 (17.64) | 55 (80.88) |
| Total | L1014F | 127 | 0.3818 | 0.6182 |  | 37 (29.13) | 71 (55.90) | 19 (14.97) |
|  | L1014S | 110 | 0.1 | 0.9 |  | 0 (0) | 22 (20.00) | 88 (80.00) |
|  | N1575Y | 139 | 0.3129 | 0.6871 |  | 10 (7.20) | 67 (48.20) | 62 (44.60) |

RR, homozygote resistant, RS, heterozygote resistant, SS, homozygote susceptible, N, total number of individuals investigated, f(R): frequency of resistant allele calculated using formula f(R) = (2 x RR +RS)/2N, f(S): frequency of susceptible allele calculated using formula f(S) = 1 – f(R). * this data is already published in previous publication Fadel *et al*. 2019

**Supplementary methods**

1. **Principal component analysis (PCA)**

This dataset used in this PCA contains 24 individuals corresponding to differentially expressed genes associated to insecticide resistance when comparing resistant Gounougou versus N’Gousso susceptible (R-S) and Control Gounougou versus N’Gousso(C-S) and 3 variables including mean expression (me), Log Fold Change in R-S (lfc1), and Log Fold Change in C-S (lfc2).

**2.1 Study of the outliers**

The analysis of the graphs does not detect any outlier.

**2.2 Inertia distribution**

The inertia of the first dimensions shows if there are strong relationships between variables and suggests the number of dimensions that should be studied.

The first two dimensions express **97.36%** of the total dataset inertia (**Figure S3**); that means that 97.36% of the individuals (or variables) cloud total variability is explained by the plane. This percentage is particularly high and thus the first plane perfectly represents the data variability. This value is greater than the reference value that equals **85.69%**, the variability explained by this plane is thus significant (the reference value is the 0.95-quantile of the inertia percentages distribution obtained by simulating 474 data tables of equivalent size on the basis of a normal distribution).

From these observations, it is absolutely not necessary to interpret the next dimensions.

An estimation of the right number of axis to interpret suggests to restrict the analysis to the description of the first 1 axis. These axes present an amount of inertia greater than those obtained by the 0.95-quantile of random distributions (67.42% against 52.72%). This observation suggests that only this axis is carrying a real information.

**Description of the dimension 1**

The dimension 1 (**Figure S4**) opposes individuals such as *SAP1* (*AGAP008051*), *CYP4G16* (*AGAP001076*), *SAP3* (*AGAP008054*) and *COEBE3C* (*AGAP005372*) (to the right of the graph, characterized by a strongly positive coordinate on the axis) to individuals such as ABCC9 ( *AGAP028128*), CYP15B1 (*AGAP005658*), CYP6AK1 (*AGAP010961*), CYP4H24 (*AGAP013490*) and CYP6P5 (*AGAP002866*) (to the left of the graph, characterized by a strongly negative coordinate on the axis).

The group in which the individual *COEBE3C* (*AGAP005372*) stands (characterized by a positive coordinate on the axis) is sharing:

- high values for the variables *lfc2* and *lfc1* (variables are sorted from the strongest).

The group in which the individuals *SAP1* (*AGAP008051*), *CYP4G16* (*AGAP001076*) and *SAP3* (*AGAP008054*) stand (characterized by a positive coordinate on the axis) is sharing:

- high values for the variable *me*.

The group in which the individuals ABCC9 (*AGAP028128*), CYP15B1 (*AGAP005658*), CYP6AK1 (*AGAP010961*), CYP4H24 (*AGAP013490*) and CYP6P5 (*AGAP002866*) stand (characterized by a negative coordinate on the axis) is sharing:

- low values for the variables *lfc1* and *lfc2* (variables are sorted from the weakest).

Note that the variable *lfc1* is highly correlated with this dimension (correlation of 0.94). This variable could therefore summarize itself as dimension 1.

1. **Classification**

The classification made on individuals reveals three clusters (**Suppl.** **Figure 5**).

Cluster **1** is made of individuals such as *AGAP012156*, *AGAP028128*, *AGAP005658*, *AGAP010961*, *AGAP004381*, *AGAP013490* and *AGAP002866*. This group is characterized by:

- low values for the variables *lfc2* and *lfc1* (variables are sorted from the weakest).

Cluster **2** is made of individuals such as *AGAP008052* and *AGAP005372*. This group is characterized by:

- high values for the variables *lfc2* and *lfc1* (variables are sorted from the strongest).

Cluster **3** is made of individuals such as *AGAP001076*, *AGAP008051* and *AGAP008054*. This group is characterized by high values for the variable *me.*

**Annexes**

dimdesc(res, axes = 1:1)

$Dim.1
$quanti
 correlation p.value
lfc1 0.9687843 8.178558e-15
lfc2 0.9419237 6.668846e-12
me 0.4435181 2.994282e-02

attr(,"class")
[1] "condes" "list "

$call
$call$num.var
[1] 1

$call$proba
[1] 0.05

$call$weights
 [1] 1 1 1 1 1 1 1 1 1 1 1 1 1 1 1 1 1 1 1 1 1 1 1 1

$call$X
 Dim.1 me lfc1 lfc2
AGAP008052 2.2405971 528.544340 5.4657404 3.8780693
AGAP005372 1.4160022 319.086263 3.4719792 3.1521134
AGAP009194 1.1850533 1619.963789 2.3224748 2.9343536
AGAP001076 1.5815219 4423.264105 2.5310292 2.7344829
AGAP009246 0.7317253 614.910470 1.6639025 2.4827594
AGAP008218 0.6757770 782.213683 1.4829998 2.3908238
AGAP002060 0.6045053 77.165498 1.5863080 2.3635702
AGAP008051 2.2852959 9586.107734 2.8612493 2.3272982
AGAP028729 0.5590072 199.111783 1.4573042 2.2742971
AGAP002204 0.6819455 28.145707 1.9540462 2.2480872
AGAP007480 0.3693736 222.561360 0.8619844 2.2359407
AGAP009468 0.5669591 108.434512 1.6065452 2.1724663
AGAP002417 0.6570875 321.307360 1.8117885 2.1642024
AGAP008206 0.3528196 320.827591 0.9163449 2.0502046
AGAP008054 1.2941659 6471.018339 2.4000544 0.6401277
AGAP012156 -1.6552564 81.325032 -2.3943321 -1.7552642
AGAP028128 -1.4250094 400.732866 -1.5508306 -2.0452614
AGAP009799 -1.4155595 343.441043 -1.4575154 -2.0907972
AGAP008214 -1.3408551 1010.246162 -1.5084935 -2.0931859
AGAP005658 -1.4263104 30.492526 -1.3212487 -2.1304893
AGAP010961 -1.4926667 2095.635845 -2.1314521 -2.5237100
AGAP004381 -1.4409033 3.610316 -0.5312009 -3.1410435
AGAP013490 -2.2530882 926.590507 -3.4140507 -3.3440244
AGAP002866 -2.7521876 17.960369 -2.8288620 -5.5525237

**Figure 5 - List of variables characterizing the dimensions of the analysis.**

res.hcpc$desc.var

Link between the cluster variable and the quantitative variables
================================================================
 Eta2 P-value
lfc2 0.8994443 3.351724e-11
me 0.8444300 3.275086e-09
lfc1 0.7959072 5.664884e-08

Description of each cluster by quantitative variables
=====================================================
$`1`
 v.test Mean in category Overall mean sd in category Overall sd
lfc1 -4.262074 -1.904221 0.6356569 0.8254602 2.213764
lfc2 -4.533921 -2.741811 0.4738540 1.1128754 2.634732
 p.value
lfc1 2.025384e-05
lfc2 5.789875e-06

$`2`
 v.test Mean in category Overall mean sd in category Overall sd
lfc2 3.740680 2.528907 0.4738540 0.5115561 2.634732
lfc1 3.064246 2.050118 0.6356569 1.2165377 2.213764
 p.value
lfc2 0.000183523
lfc1 0.002182193

$`3`
 v.test Mean in category Overall mean sd in category Overall sd
me 4.405561 6826.797 1272.196 2122.683 2285.423
 p.value
me 1.055107e-05
